# Genomic analyses reveal the genetic basis of early maturity and identification of loci and candidate genes in upland cotton (*Gossypium hirsutum* L.)

**DOI:** 10.1111/pbi.13446

**Published:** 2020-08-01

**Authors:** Libei Li, Chi Zhang, Jianqin Huang, Qibao Liu, Hengling Wei, Hantao Wang, Guoyuan Liu, Lijiao Gu, Shuxun Yu

**Affiliations:** ^1^ State Key Laboratory of Subtropical Silviculture Zhejiang A & F University Lin'an, Hangzhou; ^2^ State Key Laboratory of Cotton Biology Institute of Cotton Research of CAAS Anyang Henan China

**Keywords:** upland cotton, early maturity, genome‐wide association analysis, flowering time

## Abstract

Although upland cotton (*Gossypium hirsutism* L.) originated in the tropics, this early maturity cotton can be planted as far north as 46°N in China due to the accumulation of numerous phenotypic and physiological adaptations during domestication. However, how the genome of early maturity cotton has been altered by strong human selection remains largely unknown. Herein, we report a cotton genome variation map generated by the resequencing of 436 cotton accessions. Whole‐genome scans for sweep regions identified 357 putative selection sweeps covering 4.94% (112 Mb) of the upland cotton genome, including 5184 genes. These genes were functionally related to flowering time control, hormone catabolism, ageing and defence response adaptations to environmental changes. A genome‐wide association study (GWAS) for seven early maturity traits identified 307 significant loci, 22.48% (69) of which overlapped with putative selection sweeps that occurred during the artificial selection of early maturity cotton. Several previously undescribed candidate genes associated with early maturity were identified by GWAS. This study provides insights into the genetic basis of early maturity in upland cotton as well as breeding resources for cotton improvement.

## Introduction

Cotton is considered a model species for the study of polyploidy in plants and provides an organizational framework and phylogenetic perspective to understand the patterns and mechanisms of gene and genome evolution (Wendel *et al*., 2012). In the *Gossypium* genus, two diploid species (*Gossypium herbaceum* and *Gossypium arboreum*) as well as two allotetraploid species (*Gossypium hirsutum* and *Gossypium barbadense*) were independently domesticated in the old and new worlds (Renny‐Byfield *et al*., 2016; Wendel and Cronn, 2003; Wendel *et al*., 2012). Upland cotton (*Gossypium hirsutum* L. 2*n* = 52) has replaced the other three species and become the leading species, dominating world cotton commerce with a global yield of 75 million tons in 2010 (Wendel and Cronn, 2003).

The early maturity cotton has been widely planted in the north of China, including the cotton growing area of the Yellow River region (YRR), north of the Northwest Inland region (NIR) and the Northern Specific Early Maturity region (NSER) (Figure S1). The environment of northern China differs tremendously from that of Mesoamerica, which is the origin of *Gossypium hirsutum*. The development of early maturity cotton, which is seeded directly after wheat or rapeseed harvest, is a remarkable achievement that has helped address the grain‐cotton balance caused by population growth and loss of farmland in China. For example, between the years 1989 and 1994, ‘CRI16’, an elite variety of early maturity cotton, was widely planted in China, accounting for more than 367 million ha of planting area and greatly increasing the cotton yield on limited land. The whole growth period (WGP) of wild *Gossypium hirsutum*, which was initially domesticated at least 5000 years ago (Smith and Stephens, 1971), is actually quite long (typically >180 days); in addition, the presence of photoperiod sensitivity also hampers the use of wild *Gossypium hirsutum* in breeding programmes (Zhu and Kuraparthy, 2014). Following millennia of direct selection, early maturity of upland cotton has been greatly improved; in particular, the WGP has been reduced to approximately 140 days. In fact, breeding efforts over the past decade have decreased WGPs even further. For example, ‘Zhong213’ has a WGP of less than 105 days, with good quality and high yield (Li *et al*., 2017). Due to the breeding of early maturity cotton, the planting area has expanded to 46°N in the north of Xinjiang province. Recently, many studies have utilized next‐generation sequencing to investigate population evolution and domestication by genomic analysis in crops such as soybean (Lam *et al*., 2010; Valliyodan *et al*., 2016; Zhou *et al*., 2015), sorghum (Zheng *et al*., 2011), maize (Hufford *et al*., 2012), rice (Xun *et al*., 2012), cucumber (Qi *et al*., 2013) and tomato (Lin *et al*., 2014), which have tremendously advanced our understanding of crop domestication. However, how strong selective pressure has altered the genome of upland cotton, particularly the genetic changes underlying the adaptation to environmental changes (e.g. from tropical to temperate latitudes), and improved the early maturity remains unknown.

In this study, to gain a better understanding of the genome‐wide variations and genetic architecture of early maturity cotton, we collected a total of 436 cotton accessions with diverse WGP for genomic resequencing analysis with more than 10 Tb sequence data. In particular, the samples included 136 elite early maturity cotton accessions that have been bred in recent decades and provide a large genome variation map, providing a valuable early maturity genetic resource for future genomic‐enabled breeding.

## Results

### Genomic resequencing and variation calling

**Figure 1:**
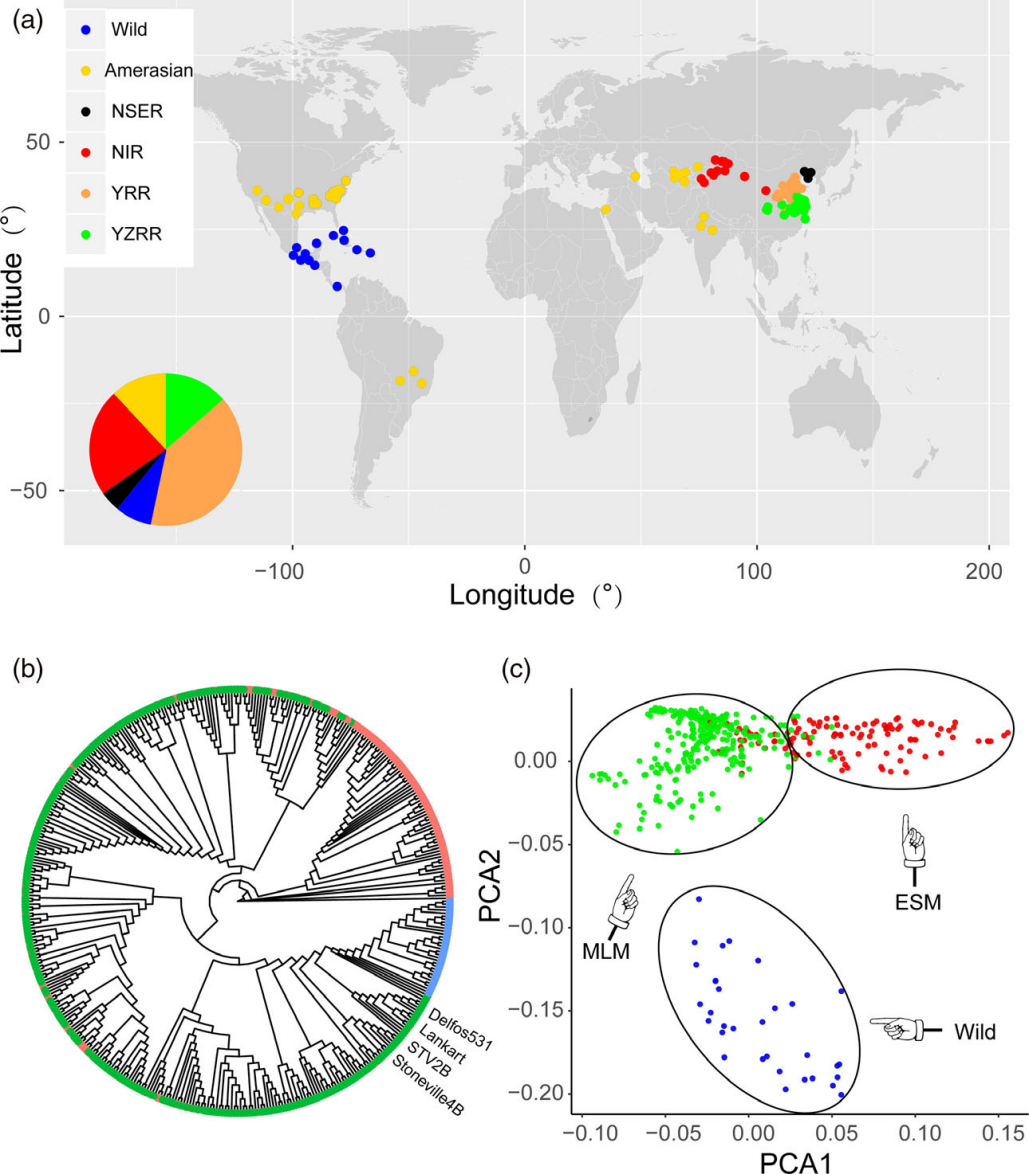
Overview of the SNP map of 436 upland cotton accessions. (a) The geographic distribution of the 436 accessions drawn using R (www.r‐project.org). Each accession is represented by a dot. Wild: 32 accessions originating from the islands in the Caribbean and Mesoamerica; Amerasian: 40 accessions primarily from central and southern Asia, America; NSER: Northern Specific Early Maturity region in China; NIR: Northwest Inland region in China; YRR: Yellow River region in China; YZRR: Yangtze River region in China. (b) Phylogenetic tree of 436 accessions inferred from 10 180 SNPs at fourfold‐degenerate sites, including three groups of wild (blue), MLM (green) and ESM (red). (c) PCA plots of 436 accessions.

A total of 436 *Gossypium hirsutum* accessions were collected worldwide for genomic sequence analysis, comprising 32 wild *Gossypium hirsutum* lines and 404 cultivars. These accessions originated from different countries and have wide geographical distribution in China, representing more than 100 years of upland cotton breeding around the world (Figure 1a and Table S1). Resequencing of the 436 upland cotton accessions on the Illumina platform generated a total of 75 149 billion paired‐end reads (10.82 Tb of sequence). The average depth for each line was approximately two‐fold greater than that of the three published genomic variation maps of *Gossypium hirsutum* (11 × versus 6.90 (Wang *et al*., 2017a), 6.55 (Ma *et al*., 2018), 5.0 (Fang *et al*., 2017b)) (Table S1). Reads were mapped to the upland cotton cultivar ‘TM‐1’ reference genome (Wang *et al*., 2019) using BWA software (Li and Durbin, 2009), with the mapping rate ranging from 62.10% to 89.30% and an average unique mapping rate of 78.23% (Table S1). After strict application of quality controls and filters, we detected 10 118 884 high‐quality SNPs and 864 132 Indels (insertion and deletions shorter than or equal to 10 bp), of which 96.64% SNPs and 97.63% Indels were located on 26 chromosomes, respectively (Tables S2, S3 and Figures S2‐S4). We then analysed the SNP position information by genomic annotation. Most SNPs (91.06%) were in intergenic regions, with only a small portion (0.66%) located in coding sequences corresponding to 19 318 genes (Figure S3). Of these, 42 052 nonsynonymous SNPs were identified among 15 006 genes, causing codon changes. The Ka/Ks (Ka, number of nonsynonymous substitutions per nonsynonymous site; Ks, number of synonymous substitutions per synonymous site) ratio was 1.82, which was higher than those found in rice (1.29) (Xun *et al*., 2012), soybean (1.61) (Lam *et al*., 2010) and pigeon pea (1.18) (Varshney *et al*., 2017), indicating positive selection during domestication. To validate the accuracy of the SNP results, we employed Sanger sequencing on 316 randomly selected SNPs from 8 accessions, along with specific‐locus amplified fragment sequencing (SLAF) based on previously reported SNP data (43 accessions with 20 206 SNPs; Su *et al*., 2016a). These methods estimated the accuracy to be 94.9% and 90.9% (Tables S4‐S6). These variants can be used for cotton functional genomic investigations in the future.

### Population structure and genetic diversity

The early maturity features of upland cotton were selected during domestication and improvement, resulting in significant variations among different populations (Figure S5). Based on their WGP, the 436 upland cotton accessions were assigned to three populations: early maturity and special early maturity cotton group (ESM), including 136 early maturity and special early maturity accessions developed in recent decades in China (WGP = 113.06 ± 10.85 days; Figure S1); medium and late maturity cotton group (MLM), including 268 medium and late maturity accessions (WGP = 134.66 ± 10.66 days); and Wild, including 32 wild *Gossypium hirsutum* lines (WGP > 180 days). To observe whether the phenotypic divergence among these three populations was supported at the genetic level, a rooted phylogenetic tree was generated using fourfold degenerate sites (4D SNPs) that represent neutral or near‐neutral variants in the complete set of 436 accessions using 10 180 SNPs (minor allele frequency (MAF) > 0.05; Figure 1b). Interestingly, the phylogenetic tree contained three major groups, corresponding roughly to the classification by phenotypic characterization. All the wild upland cotton lines clustered in an independent clade (blue), indicating that wild species exhibit not only considerable morphological but also genetic variations compared with cultivated species. Some landraces (older varieties representative of before the 1930s), such as ‘STV2B’, ‘STV4B’ and ‘Delfos531’ introduced from the US, were closer to the wild species. Similar observations were also consistent with previous research (Fang *et al*., 2017a). The remaining cultivars in the phylogenetic tree were clearly divided into separate clusters corresponding to ESM and MLM. Similar results were found via principal component analysis (PCA; Figure 1c). We then analysed the structure of the ancestral properties in each accession by increasing *K* (the number of populations) from 2 to 10 (Figure S6). The best suitable number of subpopulations was set to 2 by ‘chooseK’ packages (Raj *et al*., 2014) and revealed a very distinct divergence between wild *Gossypium hirsutum* and cultivated *Gossypium hirsutum*. However, for *K* = 3, the cultivar group revealed extensive admixture between ESM and MLM. Furthermore, to evaluate the genetic diversity among all samples, we further quantified variations in the nucleotide diversity and linkage disequilibrium (LD) for all three populations. Nucleotide diversity (*π* value) was significantly higher for the Wild population (1.01 × 10^−3^) than for the cultivars (MLM: 0.71 × 10^−3^ and ESM: 0.68 × 10^−3^; Figure S7). Earlier reports have suggested the presence of a high LD in cotton genomes (Fang *et al*., 2017b; Ma *et al*., 2018; Wang *et al*., 2017a). We used PLINK software (Purcell *et al*., 2007) to calculate correlation coefficient values (*r* ^2^) of alleles to measure the LD decay rate in the three populations. The cultivars (MLM: 288 kb and ESM: 320 kb) exhibited higher LD decay rates than the wild group (158 kb) when decreased to half its maximum value (Figure S8). The results of phylogenetic, PCA, population structure and LD analyses revealed a very distinct divergence between wild *Gossypium hirsutum* and cultivated *Gossypium hirsutum*, consistent with previous reports (Fang *et al*., 2017a; Wang *et al*., 2017a). We also found the ESM population showed a lower nucleotide diversity and higher LD and was enriched within a small clade of the phylogenetic tree, suggesting that ESM originated as an improved form of MLM but not highly structured and thus were suitable for genome‐wide association analysis (GWAS) (Yano *et al*., 2016). This interpretation is also supported by the pedigree (Figure S9).

### Adaptation changes and increasing early maturity during domestication

Upland cotton originated in tropical and subtropical regions (Wendel *et al*., 2012) but can now be planted in Xinjiang, northern China (46°N). Selective breeding for earlier maturity may have caused the genetic structure to diverge during domestication and improvement. To address how the cotton adapts to the low‐temperature region, northern China, we first calculated population differentiation using *F* statistics. The differentiation between groups was further evaluated based on the *F*_ST_ value. The highest population differentiation estimate (*F*_ST_) was observed between the wild lines and cultivars, with a pairwise *F*_ST_ of 0.31 (MLM) and 0.32 (ESM) (Figure S7). In comparison, the *F*_ST_ value (0.05) of the MLM and ESM populations was lower (Figure S7). Our results again confirm that the Wild population is the ancestral population and that ESM experienced adaptive changes over a long period of domestication and artificial selection in the breeding history.

**Figure 2:**
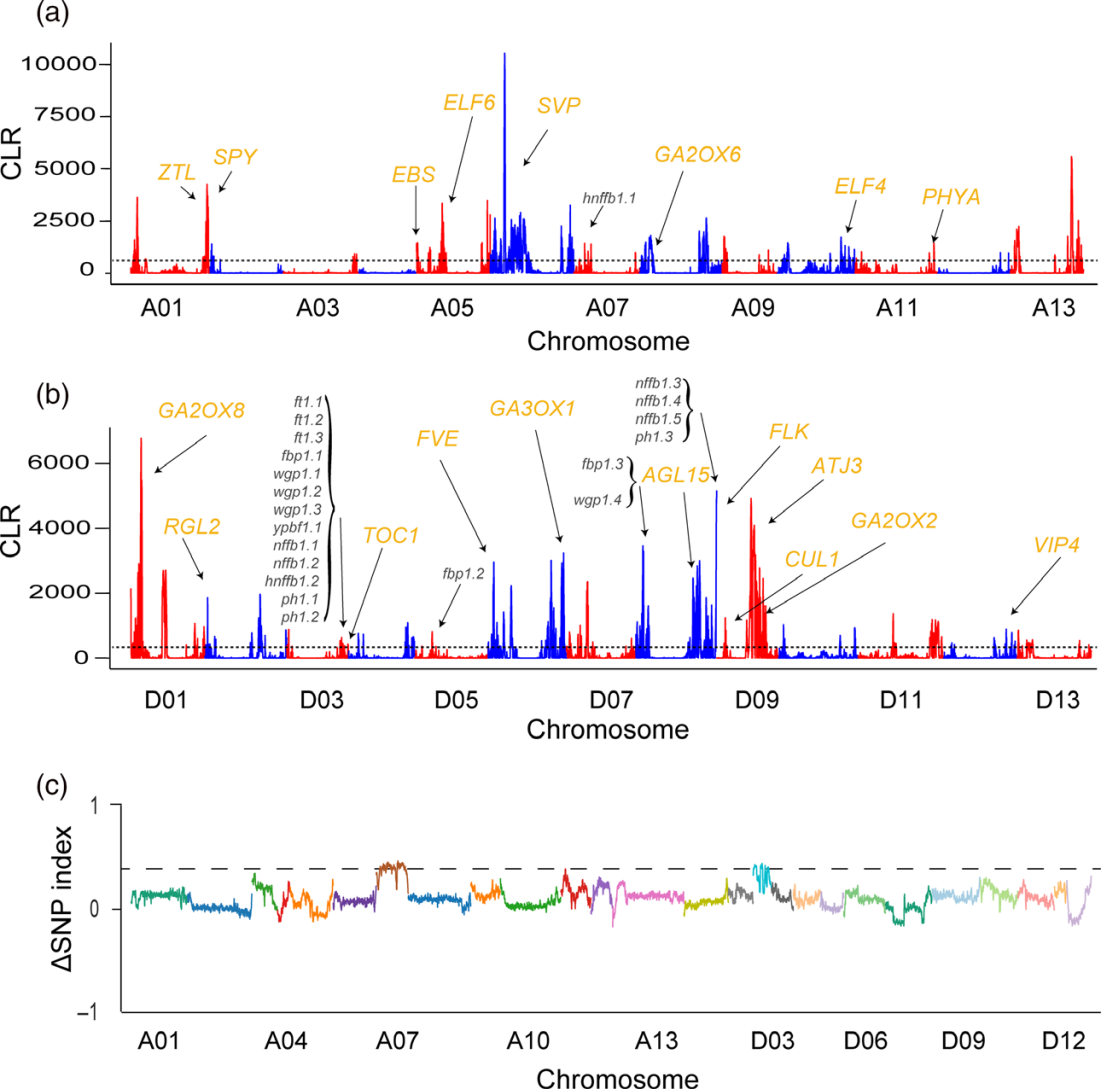
Candidate genomic regions under selection in early maturity cotton. (a,b) Selection sweeps in the A subgenome (At) (a) and the D subgenome (Dt) (b); the *y*‐axis represents the composite likelihood ratio, and the *x*‐axis represents the chromosome numbers. The solid black line indicates the cut‐off for the top 5% of windows. The 21 early maturity‐related trait QTL hotspots that overlap with selection sweeps are shown in black for each chromosome. Gibberellin catabolism (*GA2OX6*: *Ghir_A08G004420*; *GA2OX8*: *Ghir_D01G006870*; *GA3OX1*: *Ghir_D06G021240*; *GA3OX1*: *Ghir_D06G021240*; *GA2OX2*: *Ghir_D09G008270*) and flowering time (*SPY*: *Ghir_A01G019780*; *EBS*: *Ghir_A05G017710*; *ELF6*: *Ghir_A05G030300*; *SVP*: *Ghir_A06G014970*; *ELF4*: *Ghir_A10G024000*; *PHYA*: *Ghir_A11G028330*; *RGL2*: *Ghir_D02G006710*; *TOC1*: *Ghir_D03G010390*; *FVE*: *Ghir_D06G007770*; *ZTL*: *Ghir_D08G002610*; *AGL15*: *Ghir_D08G015850*; *FLK*: *Ghir_D08G023290*; *ATJ3*: *Ghir_D09G010620*; *CUL1*: *Ghir_D09G006690*; *VIP4*: *Ghir_D12G019520*) candidate genes are marked in orange. (c) *x*‐axis represents the position of 26 chromosomes and *y*‐axis represents the Δ SNP index (subtracting the SNP index of the early flowering bulk population from that of the late flowering bulk population).

Second, to identify genomic regions affected by selection that were important for adaptation during domestication and improvement, we examined whole‐genomic‐region signals of selective sweeps using a site frequency spectra (SFS)‐based method (Pavlidis *et al*., 2013; Figure 2a, b). A total of 357 selective sweeps were detected (Table S7), covering 4.94% (112 Mb) of the upland cotton genome and harbouring 5184 genes (Table S8). Among the sweep regions, 16.38% were intergenic, implying a potential regulatory role in domestication and breeding. Similarly, a study of maize inbred lines showed that many putative sweep regions were located in nongenic regions by CLR analysis (Jiao *et al*., 2012). Functional classification of the genes presents in the sweep regions showed that their products are predicted to be involved in four significantly enriched biological process GO terms, namely, flowering time, hormone catabolism, defence responses and ageing (false discovery rate [FDR] < 0.01; Table S9). Early maturity is well known to be accompanied by early senescence during the development of early maturity cotton (YU *et al*., 2005). The ageing process category included multiple genes (*WRKY22*, *VIN2*, *PUB44*, *SAG21*, *UBA2c* and *SWEET15*) (Gao *et al*., 2016; Kim *et al*., 2008; Veyres *et al*., 2008; Vogelmann *et al*., 2012; Yang *et al*., 2011b; Zhou *et al*., 2011) known to be highly expressed during leaf senescence in early maturity cotton (Lin *et al*., 2013). Hormone signalling also plays diverse and critical roles during plant development, and many well‐known genes were significantly enriched in the hormone catabolic process category (Table S9). For example, *CKX3* encode proteins with sequences similar to that of cytokinin oxidase and plays an important role in regulating flower organ development (Ding *et al*., 2015). *GA2OX2*, *GA2O*X6 and *GA2O*X8 participate in the inactivation of gibberellin and affect flowering behaviour (Rieu *et al*., 2008; Schomburg *et al*., 2003). Notably, flowering time (FT) is an important early maturity‐related trait in cotton and has also been well characterized in soybean (Zhang *et al*., 2015). Several candidate FT genes within sweep regions were homologous to members of the photoperiod pathway (*CUL1*, *TOC1*, *PHYA*, *ZTL* and *ELF4*), vernalization pathway (*VIP4*), autonomous pathway (*FVE* and *FLK*), GA signalling (*SPY* and *RGL2*), and to integrators and downstream genes (*ATJ3*, *AGL15*, *AGL24*, *EBS* and *ELF6*; Grover *et al*., 2015; Komeda, 2004; Ratcliffe and Riechmann, 2002; Table S9). These biological processes suggest potential roles in the improved early maturity of domesticated cotton.

Linkage analysis is an effective tool for identifying genes that control some of the most important morphological changes during domestication and improvement (Doebley *et al*., 2006). Over the past decade, many quantitative trait loci (QTLs) related to early maturity traits have been identified (Guo *et al*., 2008; Jia *et al*., 2016; Kushanov *et al*., 2017; Li *et al*., 2013a; Li *et al*., 2016; Li *et al*., 2017; Su *et al*., 2016b; Zhang *et al*., 2016). To assess whether the sweep regions associated with domestication colocalize with loci known to control early maturity‐related traits, we overlapped selection sweeps with the locations of known QTL hotspots. We detected a total of 21 QTLs in sweep regions, which likely played an important role during the remarkable improvement of maturity during long‐term selection (Figure 2a, b and Table S10). Interestingly, QTL hotspot regions on chromosome D03 were overlapped in selection sweeps. In this region, an ortholog of *TOC1* (*Ghir_D03G010390*), which influences flowering time through the circadian clock‐regulated photoperiod pathway, promotes the expression of FLOWERING LOCUS T *(FT)* and SUPPRESSOR OF OVEREXPRESSION OF CO 1 *(SOC1)* by controlling CONSTANS *(CO)* (Jung and Müller, 2009). Moreover, previous studies have demonstrated that *Ghir_D03G010390* expression differs significantly between early and late maturing varieties (Li *et al*., 2017). Therefore, *Ghir_D03G010390* may be a key gene related to the control of flowering time during the breeding process.

We next chose an F_2_ population and performed bulked segregant analysis (BSA) using resequencing data from extreme FT lines to further verify selection sweeps related to early maturity (Table S11). The notable variations in FT among modern domesticated cotton are related to photoperiod sensitivity. The frequency distributions showed continuous variations, indicating that FT is a quantitatively inherited trait (Figure S10). Interestingly, three regions associated with FT have strong signals (A07: 20.53–21.60 Mb; D03: 35.49–36.49 Mb and 39.17–40.29 Mb) overlapped with selection sweeps that had experienced artificial selection during improvement (Figure 2c). This result verifies the accuracy of the identified selection sweeps associated with the domestication of early maturity in cotton.

### GWAS for early maturity‐related traits in upland cotton

From 2015 to 2017, seven quantitative traits for early maturity, comprising FT, the period from first flower blooming to first boll opening (FBP), WGP, yield percentage before frost (YPBF), node of the first fruiting branch (NFFB), height of the node of the first fruiting branch (HNFFB) and plant height (PH), were investigated at three locations. Significant variation was observed for seven maturity‐related traits among the 355 upland cotton accessions (Figure S11). Pearson’s correlation coefficient analysis showed a significant negative correlation between YPBF and the other traits, all six of which showed significantly positive pairwise correlations (Figure S12). ANOVA revealed that early maturity‐related traits presented significant environmental and genetic effects (*P* < 0.001; Table S12). A broad sense heritability analysis was performed, and all traits ranged from 0.67 (WGP) to 0.79 (FT).

GWAS performed using 355 accessions from eight environments further identified a total of 307 significant SNP loci that were mainly distributed on chromosomes A01, A02, A03, A05, A06, A07, D01, D03 and D05 (Table S13 and Figures S13‐S20). Of all these significant loci, six (rsD03_37996318, rsD03_37952328, rsD03_38191576, rsD03_38175272, rsD03_38370420 and rsD03_39122594) shared more than four traits, indicating a genetic basis for pleiotropism that made it possible to simultaneously improve multiple early maturity traits during breeding. Notably, two distinct enrichment regions were located on chromosome A05 and chromosome D03, which accounted for more than 88.92% (273) of the loci.

**Figure 3:**
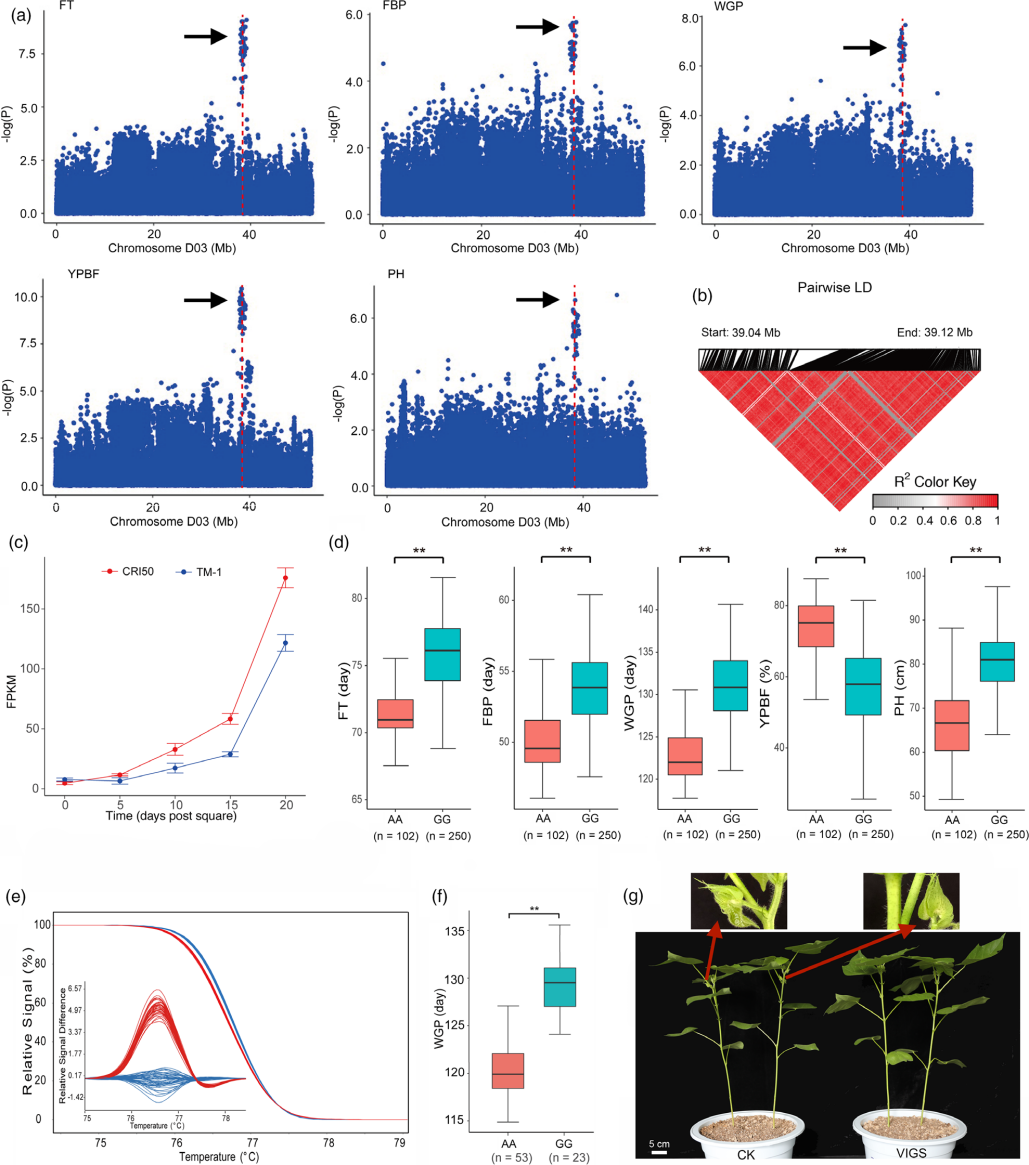
GWAS for early maturity‐related traits and identification of the candidate gene on chromosome D03. (a) Manhattan plots for flowering time (FT), period from the first flower blooming to the first boll opening (FBP), whole growth period (WGP), yield percentage before frost (YPBF) and plant height (PH) on chromosome D03; Arrowheads indicate the strongly loci rsD03_39122594 associated with the candidate gene *Ghir_D03G011310*. (b) LD heat map for the 82.17 kb long candidate region. The pairwise LD between the SNP markers is indicated as *D*′ values, where red indicates a value of 1 and grey indicates 0. (c) Expression profiles of *Ghir_D03G011310*. The x‐axis represents developmental stages (0, 5, 10, 15 and 20 DPS), and the y‐axis indicates the relative expression levels as determined by RNA‐seq. The error bars indicate standard deviation of three biological replicates. (d) Box plots for FT, FBP, WGP, YPBF and PH between two haplotypes mentioned above (** *P* < 0.01). (e) HRM analysis for SNP (rsD03_39122594) in recombinant inbred line population. The axis of the outside is original melting curves; the axis of the inside is melting curves after logarithm. Red and blue curves correspond to favourable alleles (A) and unfavourable alleles (G), respectively. (f) Box plots for two haplotypes in whole growth period at recombinant inbred line population mentioned above (** *P* < 0.01). (g) VIGS of *Ghir_D03G011310* in early maturity cotton ‘CRI50’. ‘CRI50’ treated with an empty vector were used as a control group. Red arrows indicate the squares and fruit branches.

Previous studies have indicated that chromosome D03 is rich in QTLs for early maturity traits (Jia *et al*., 2016; Li *et al*., 2013a; Li *et al*., 2017; Su *et al*., 2016b). In the present study, 43 significantly associated SNPs spanned a region of approximately 3.7 Mb on chromosome D03 (between 36.68 and 40.38 Mb). Of these, 88.37% (38) loci overlapped with our previously mapped early maturity QTLs and selection sweeps. These results strongly suggest that chromosome D03 is a major region and has potential functional genes related to early maturity. Interestingly, two associated SNPs lie within the most significant haplotype block, which is 82.17 kb long and contains three genes (Figure 3a, b). The RNA‐seq data showed that *Ghir_D03G011310* had higher expression levels in the early maturity variety ‘CRI50’ than in the late maturity variety ‘TM‐1’ compared with the other two genes during flower development from 0 to 20 DPS (Figure 3c and Figure S21). Gene annotation analysis indicated that *Ghir_D03G011310* encodes a cysteine protease, the closest known homologue in *Arabidopsis thaliana* of which is *CEP1*, which is expressed specifically in the tapetum and is involved in pollen development (Zhang *et al*., 2014). Furthermore, quantitative reverse‐transcription PCR (qRT‐PCR) showed that the expression of *Ghir_D03G011310* in developing stages at three leaf growth stages and four leaf growth stages was significantly higher in the early maturity varieties (‘Zhong213’ and ‘CRI50’) than in the late maturity varieties (‘NDM8’ and ‘TM‐1’; *P* < 0.01; Figure S22). The SNP (rsD03_39122594) was located 1810 bp upstream of the start codon of *Ghir_D03G011310* (Figure S23), which was found to have the strongest association with FT, WGP, YPBF and PH (average *P* value = 4.46E‐08). Varieties carrying the HapA (A allele) exhibited earlier maturity than carrying of the HapB (G allele) (Figure 3d). To confirm the practical utility of this locus, high‐resolution melting (HRM) analysis was used to genotype HapA and HapB in two recombinant inbred line (RIL) populations generated by crossing the early maturity line with the late maturity line from a previous report (Jia *et al*., 2016; Li *et al*., 2017; Figure 3e, f and Figure S24). The HRM assay showed that the mean WGP in the two RIL lines with the A allele was significantly shorter than that in the lines with the G allele, which is consistent with the GWAS results (Figure 3d). We further validated the function of *Ghir_D03G011310* through virus‐induced gene silencing (VIGS) in early maturity cotton ‘CRI50’. The fruit branches or squares have not been observed in the silenced plants. However, the CK plants have yielded fruit branches with squares at the same growth status as shown in Figure 3g. And as shown in Figure S25, the drastically reduced expression of the *Ghir_D03G011310* in the VIGS plants demonstrated that the gene had been successfully knocked down. We then analysed the nucleotide diversity, *F*_ST_ values and Tajima’s *D* for the strongly LD block region (82.17 kb) that mentioned in the Figure 3b contained *Ghir_D03G011310* in the flanking 150 kb region. Interestingly, it clearly showed that ESM group had lowest diversity than wild and MLM group and *F*_ST_ values of ESM versus MLM was significantly higher than the flanking region (Figure S26). The Tajima’s *D* value in this strongly LD block region was −0.81 and biased from the balance. Thus, from the above results, we inferred that *Ghir_D03G011310* is a previously undescribed gene that contributes to early maturity in cotton and involved in artificial selection in cotton.

**Figure 4:**
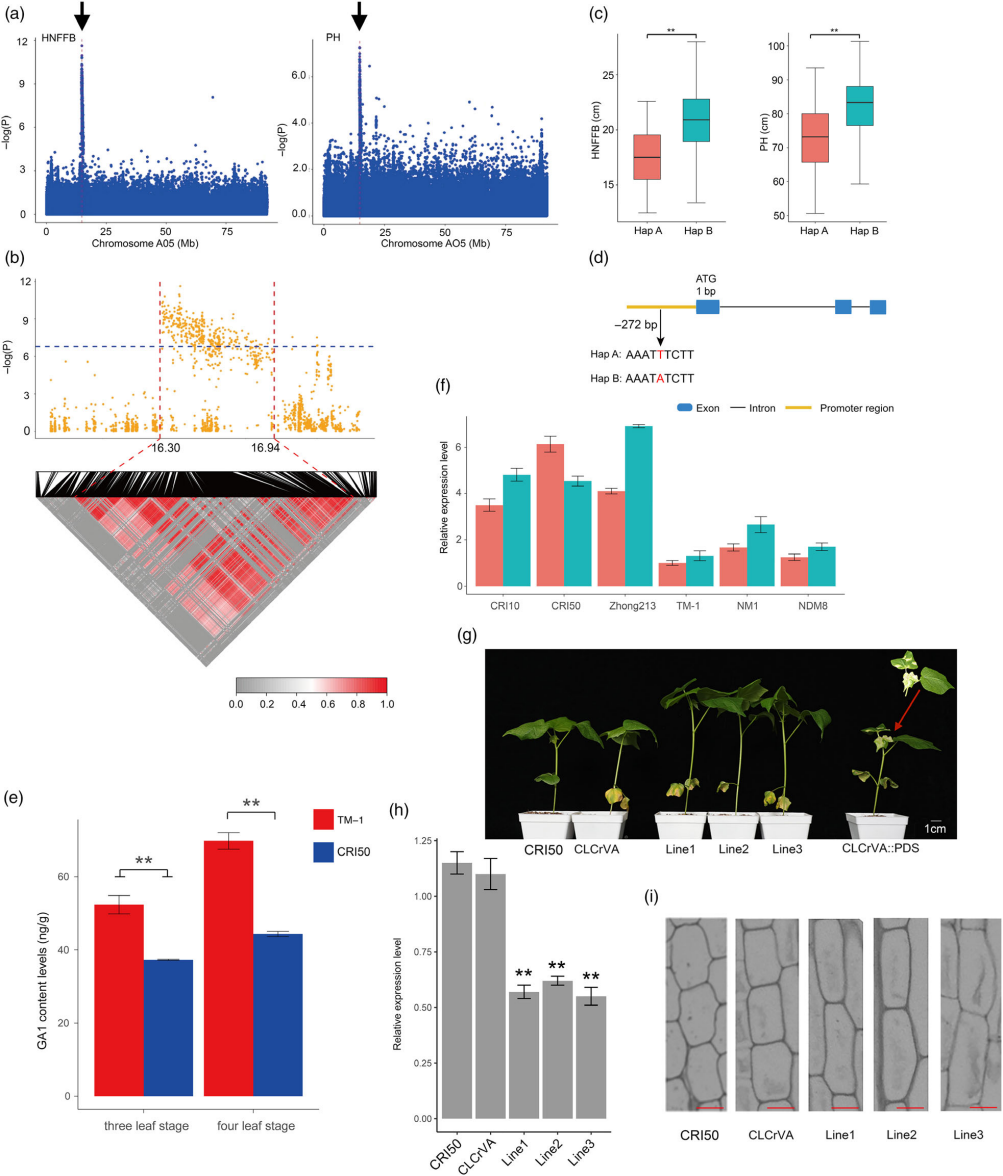
GWAS for early maturity‐related traits (HNFFB and PH) and identification of the candidate gene on chromosome A05. (a) Manhattan plots for height of the node of the first fruiting branch (HNFFB) and plant height (PH) on chromosome A05; Arrowheads indicate the strongly loci rsA05_16520008 associated with the candidate gene *GhGA2OX8*. (b) Local Manhattan plot (top) and LD heat map (bottom). The candidate region lies between the red dashed lines. (c) Box plots for rsA05_16520008 with two haplotypes in HNFFB and PH mentioned above (** *P* < 0.01). (d) Gene structure of *GhGA2OX8* and the polymorphism in two haplotypes. (e) GA_1_ content measured in the apical bud of ‘CRI50’ and ‘TM‐1’ plants. (f) Comparison expression levels of *GhGA2OX8* at stem apexes during three (red) and four (green) leaf stage. (g) Phenotype of VIGS *GhGA2OX8* in early maturity cotton ‘CRI50’. ‘CRI50’ treated with an empty vector were used as a control group. (h) Expression level of *GhGA2OX8* in ‘CRI50’, empty control and VIGS lines. (i) Longitudinal section from the first internode of ‘CRI50’, empty control and VIGS lines, scale bar is 25 μm.

We then focused on chromosome A05 (Figure S13). These loci exhibited pleiotropic associations with PH and HNFFB. We identified 75.57% (232) peaks that were associated with the phenotypic variation. Notably, 237 loci were tightly linked within the candidate region (Table S14), a 16.30–16.94 Mb (630.69 kb) segment on chromosome A05 (Figure 4a, b) containing 57 candidate genes. Previous studies have shown that a majority (93%) of GWAS loci are located in nongenic regions and have regulatory functions (Maurano *et al*., 2012). However, in this 630.69 kb region, the annotated genetic variants of approximately 40% (134) of the identified loci were nonintergenic. These loci may directly control the relevant biological pathways involved in early maturity that are linked to causal polymorphisms in nearby genes. SNP rsA05_16453277 (G/T) was in the 3′ UTR regions of *Ghir_A05G017290*. A total 125 accessions exhibiting the T/T homozygous allele showed a shorter PH and HNFFB than those with the G/G homozygous allele (Table S15 and Figure S27). In our transcriptome analyses, *Ghir_A05G017290* was more highly expressed in the early maturity variety ‘CRI50’ than the late maturity variety ‘TM‐1’ at the flowering development stage (Figure S28). In addition, *Ghir_A05G017290* was a homologue of *OsbHLH068* that encodes an MYB transcription factor in rice. Heterologous overexpression of *OsbHLH068* in *Arabidopsis* regulates the expression of genes involved in the control of flowering time (Chen *et al*., 2017). These results suggest that *Ghir_A05G017290* might determine early maturity in cotton.

Early maturity cotton is a short and compact plant (Figures S5 and S7). PH and HNFFB are very important early maturity traits that correlate significantly with FT, FBP, WGP and YPBF (Figure S12), and previous studies have also shown that maturity and plant architecture genes exhibit a strong correlation (Li *et al*., 2013a; Li *et al*., 2017). In the present study, *GhGA2OX8* (*Ghir_A05G017390),* was significantly associated with PH and HNFFB in the promoter region of the 630.69 kb tight‐linkage region (Figure 4c, d and Table S15). Interestingly, we additionally observed that another gene, *Ghir_D05G017200*, also homologous to *GhGA2OX8*, was identified on chromosome D05 associated with HNFFB (Figure S29). Notably, GA biosynthesis and the role of GA signalling in controlling plant height have been investigated in rice, wheat and maize (Wang *et al*., 2017b). *GhGA2OX8* is annotated as encoding a gibberellin 2‐oxidase involved in the biosynthesis of gibberellin. Overexpression *GA2OX8* in *Arabidopsis* and tobacco has been shown to decrease gibberellin levels and create dwarf varieties (Schomburg *et al*., 2003). Interestingly, we discovered that the early maturity variety ‘CRI50’ (PH = 52.43 ± 6.88 cm) carrying the A allele contained significantly less active GA_1_ than the late maturity variety ‘TM‐1’ with the G allele (PH = 76.55 ± 8.49 cm; Figure 4e). The qRT‐PCR results indicated that *GhGA2OX8* expression was higher in apical buds in early maturity varieties (PH = 59.25 ± 5.69 cm) than in those in late maturity varieties (PH = 87.96 ± 4.39 cm; Figure 4f). Overexpression of the *GhGA2OX8* in *Arabidopsis* resulted in dwarf phenotypes compared with the wild type (Figure S30), and the height of each line was presented in Table S16. Furthermore, the resulting VIGS lines had significantly higher and drastically reduced expression of the *GhGA2OX8* (Figure 4g, h) with parenchyma cells showing increased length compared with ‘CRI50’ and CLCrVA (empty vector; Figure 4i and Figure S31). From the above results, we inferred that *GhGA2OX8* has a potential role in controlling cotton stature in the future.

### Elite alleles for pyramid breeding in ESM

**Figure 5:**
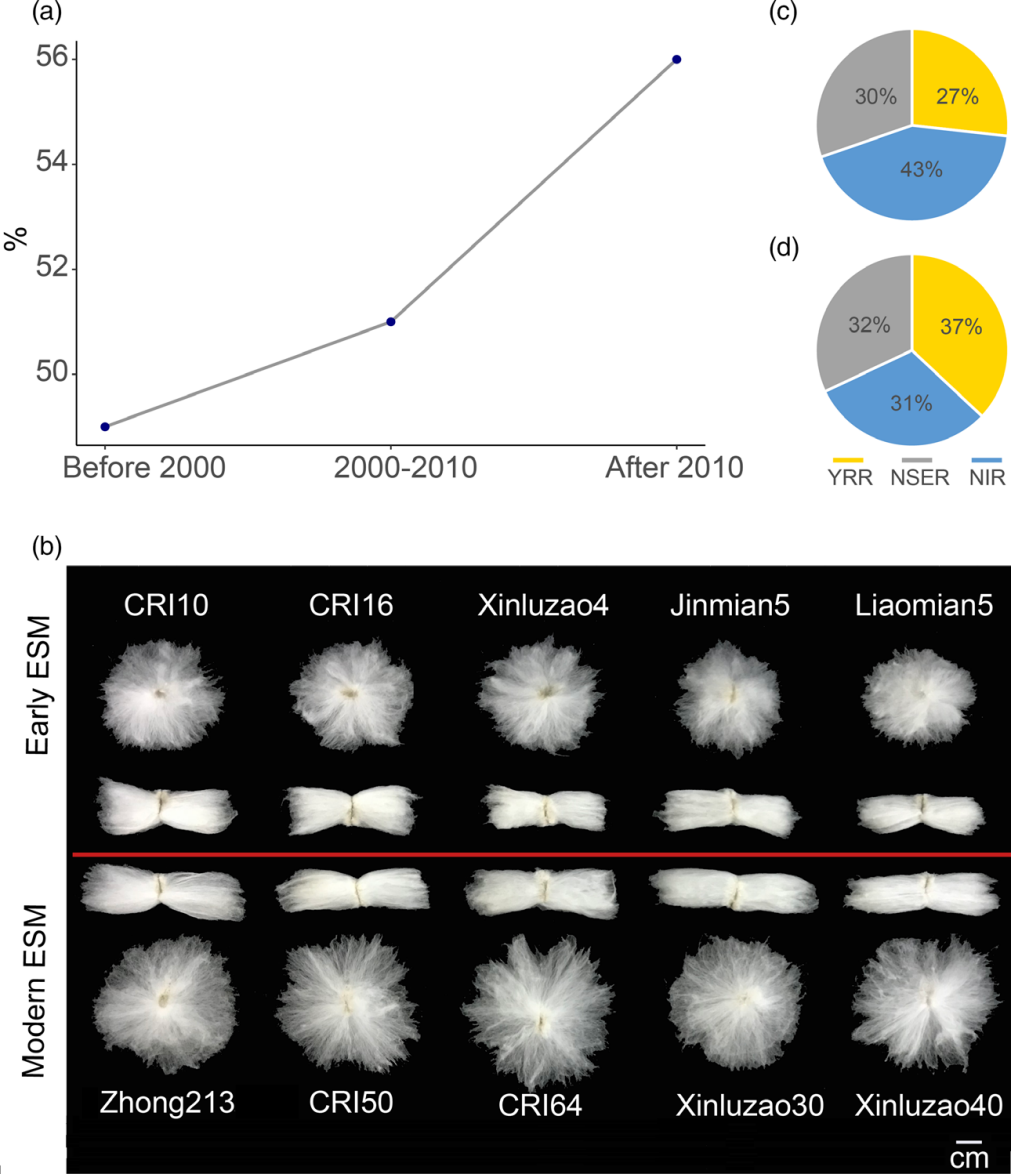
Elite alleles distributions for the fibre quality and yield traits in the early maturity cotton. (a) Percentages of elite alleles in early maturity cotton cultivars released before 2000s, 2000s–2010s and after 2010s. (b) Representative images of individual seeds with attached fibre between early ESM and modern ESM. (c, d) Percentages of elite alleles for fibre quality and yield traits in Yellow River region (YRR), Northern Specific Early Maturity region (NSER) and Northwest Inland region (NIR) in China, respectively.

We selected 136 early maturity accessions (Table S1) and collected 27 elite loci associated with yield and fibre quality from previous studies (Fang *et al*., 2017b; Wang *et al*., 2017a; Table S17). Based on the release time, the 136 ESM lines used in this study were divided into three groups: before the 2000s, 2000s–2010s and after 2010. Analysis of the distribution of elite alleles indicated that the percentages of total elite alleles have gradually increased at the selected 27 SNP loci to 49.6%, 51.2% and 56.2% (Figure 5a).

## Discussion

Here, we report a comprehensive genome variation map of upland cotton by deep resequencing that is more comprehensive than recent publications (436 versus 352 (Wang *et al*., 2017a), 318 (Fang *et al*., 2017b) and 419 (Ma *et al*., 2018) accessions). The large nature population represents a core cotton germplasm from the early 1900s to 2010s. Although 79% of the accessions are cultivated in different ecological cotton growing areas of China: Yellow River region, Yangtze River region, north of the Northwest Inland region and the Northern Specific Early Maturity region, we also included many early landraces such as ‘King’, ‘Deltapine’, ‘Stoneville’ and ‘Acala’ from the US cotton belt. Most importantly, we collected 136 elite early maturity breeding lines and cultivars bred in recent decades, of which 48 accessions having a WGP within 110 days in particular. The phenotypic diversity of early maturity‐related traits was more abundant (Table S12 and Figure S11), which suggested that the panel contained a greater variety of elite alleles associated with early maturity‐related traits than previous GWAS populations (Fang *et al*., 2017b; Ma *et al*., 2018; Wang *et al*., 2017a). In addition, GWAS in cotton based on whole‐genome resequencing has been focused more on fibre quality, yield and disease resistance (Fang *et al*., 2017b; Ma *et al*., 2018; Wang *et al*., 2017a), but the genetic mechanisms of early maturity‐related traits are unclear. Previous reports have indicated that mutation and favourable allele accumulation are probably two major routes to improve important agronomic traits in crops (Moose *et al*., 2004). Our results provide evidence for the latter hypothesis in the GWAS, in which we performed the first genome‐wide association analysis for seven early maturity‐related traits by resequencing.

After strict quality controls and filters of variations, we detected more high‐quality SNPs used genomic analysis (10 118 884 versus 8 621 073 (Fang *et al*., 2017b); 7 497 568 (Wang *et al*., 2017a) and 3 665 030 (Ma *et al*., 2018)) than previous research. The number of SNPs as well as the nucleotide diversity significantly decreased throughout the domestication process from wild to cultivated cotton (Figure S7), consistent with previous studies (Fang *et al*., 2017a; Wang *et al*., 2017a). Our data revealed that 2 002 869 private SNPs in wild cotton were not polymorphic in the modern cultivar (ESM and MLM). Previous studies have hypothesized that modern plant breeding reduces genetic diversity and jeopardizes future crop improvement (Li *et al*., 2013b). Although this concept seems correct for the majority of crop species (Lin *et al*., 2014; Qi *et al*., 2013; Valliyodan *et al*., 2016), our data showed limited effects of breeding on the reduction in nucleotide diversity between the MLM and ESM group. We found that the ESM gene pool harboured a high proportion of the SNPs (87.72%) presented in the MLM group and show a reduction in nucleotide diversity of 2.4%, possibly due to the shorter time for artificial selection from medium and late maturity to early maturity. In addition, the phylogenetic results were corresponding roughly to the whole growth period characteristics, whereas contrast with a previous study in other crops, in which the populations were structured geographically (Lam *et al*., 2010). However, similar result in cotton has been reported by Ma *et al*. (2018). These observations suggest that upland cotton has a relatively lower genetic diversity due to gene introgression, interspersed introduction or hybrid breeding among these very closely related individuals and narrow the introduction of resources from the US in the first 30 years of the twentieth century (Huang, 2007; Huang *et al*., 2017).

In this study, a gibberellin biosynthesis‐related pathway gene *GhGA2OX8* associated with early maturity have been found by GWAS analysis. The overexpression of *GhGA2OX8* showed consistent results, as observed in *GA2OX8*, revealing conserved functions between *GhGA2OX8* and *GA2OX8* in reducing plant height. Virus‐induced gene silencing cotton has significantly increased plant height when the expression of the *GhGA2OX8* was suppressed. During the ‘Green Revolution’ of the 1960s and 1970s, the genetic manipulation of dwarfing genes led to the development of semidwarf rice and wheat varieties with improved lodging resistance and yield (Khush, 2001). In recent years, decreases in the cotton planting area in China have left cotton breeders struggling to maximize the yield from limited land. Increasing the planting density to raise the yield is much more effective than increasing yields from individual plants (Duvick, 2005). Planting early maturity cotton, which increases the lint yield by increasing the boll number per unit area, is an effective method to improve the cotton plant density and satisfy demand. In the present study, the *GhGA2OX8* showed excellent potential for improving the plant architecture. Through the improvement of plant architecture by *GhGA2OX8*, which planting at a high plant density and make the canopy more compact, it could contribute to cotton yield under high‐density cultivation in fields and be a selection target for the genetic improvement of early maturity in cotton.

Although our genomic analyses identified many genes previously known to influence early maturity, we also identified a new candidate gene *Ghir_D03G011310* was greatly upregulated during flower development from 0 to 20 DPS and more highly expressed in the early maturity variety by transcriptome and qRT‐PCR. VIGS of *Ghir_D03G011310* in early maturity cotton also resulted in delayed formation of fruit branches and squares. Furthermore, with the widely application of next‐generation sequencing technology, the density of molecular markers is increasing. Investigating the artificial selection history of a specific gene is one way to decipher the history of crop and its impact on humans (He *et al*., 2015). We also found *Ghir_D03G011310* is an artificial selection gene. The genetic diversity of ESM was lower than that of MLM and wild for the genomic regions of *Ghir_D03G011310* and proved by *F*_ST_ and Tajima’s *D* test. This artificially imposed selection pressure on the expression of *Ghir_D03G011310* could be an important factor affecting the observed difference in early maturity in cotton, because the varieties carrying the HapA resulted in improved early maturity than carrying of the HapB. Therefore, the *Ghir_D03G011310* should be useful for improving early maturity in upland cotton breeding via a molecular design approach, so as to further ensure stable cotton production.

Early maturity has always had a negative genetic correlation with yield and fibre quality. The early maturity breeding programme was launched in China in the 1970s; however, the yield and fibre quality of early maturity cotton have been greatly improved (Figure 5b). We also found that 22.1% (1146) of the genes were differentially expressed during ovule and fibre development in the selected regions (Table S18). This finding demonstrates that with an increase in early maturity through breeding, fibre yield and quality are also altered by artificial selection, an effect known as ‘domestication syndrome’ (Doebley *et al*., 2006). For example, the early stage of ‘CRI10’ has a poor yield and fibre quality compared with the modern early maturity variety ‘Zhong213’ (Li *et al*., 2017). How have the elite alleles associated with yield and fibre quality changed within early maturity accessions during the four decades of breeding development? To address this question, we found that with improvement of early maturity in cotton, elite loci associated with yield and quality became increasingly popular and were gradually integrated into the breeding process over time. Furthermore, the percentages of elite alleles related to fibre quality were much greater in the NIR than in the YRR and NESR populations, whereas the opposite trend was observed for yield (Figure 5c, d). This phenomenon was likely caused by different origins and regional breeding objectives.

In summary, our future work will not only examine particular loci (genes) but also characterize the regulatory networks/pathways underlying early maturity by utilizing functional genomic methodologies such as genome editing and genetic transformation to validate the effects of these candidate genes on the genetic improvement of modern cultivars.

## Experimental procedures

### Plant materials and resequencing

A total of 436 diversity upland cotton accessions were collected from multiple countries with a wide geographic distribution, including China, the United States, Uzbekistan, India, Brazil and other countries. The panel including 356 accessions that were newly produced in this study and 80 resequencing data sets that were previously analysed by Fang *et al*. (2017a); Wang *et al*. (2017a). Detailed information on the 436 accessions is listed in Table S1. The geographic distribution of upland cotton accessions was visualized with the R packages of ‘ggplot2’ and ‘maps’ (Brownrigg, 2013; Wickham, 2016). Young leaves were collected 4 weeks after planting and quickly frozen in liquid nitrogen for sequencing. Total DNA was extracted using the CTAB method (Paterson *et al*., 1993). For each accession, at least 5 μg of genomic DNA was used to construct paired‐end sequencing libraries.

### Identification of variation and filtering

All paired‐end sequence reads were mapped to the *Gossypium hirsutum* cv. TM‐1 reference genome (Wang *et al*., 2019) using BWA software with default parameters (Li and Durbin, 2009). Only reads with unique mapping position in the TM‐1 reference genome and mapping quality value greater 30 were retained in BAM format by SAMtools (Li *et al*., 2009). The Picard programme was used to sort mapping results from name order into coordinate order and mark the PCR reads that were duplicated during library construction or sequencing. Additionally, we improved the alignment performance by realignment of reads around Indels from the BWA mapping results with the IdnelRealigner package in the Genome Analysis toolkit (GATK) (McKenna *et al*., 2010). SNP and Indel detection were performed using bcftools (Li, 2011) and GATK software. High‐quality SNPs and Indel variations were obtained according to the following criteria. (a) Only concordant sites identified by GATK and bcftools with the SelectVariants packages were retained. (b) SNPs within the 5‐bp range of Indels were filtered out. (c) The SNP quality value should be greater than 30. (d) SNPs and Indels with a MAF < 1% and missing rate < 10% were discarded. (e) The average sequencing depth had to be greater than 5 × and less than 30×. (f) Insertions and deletions with a maximum length 10 bp were taken into account. The annotation information of variants was obtained by ANNOVAR (Wang *et al*., 2010).

### SNP validation

Two methods were used to validate the resequencing accuracy and quality. (1) We randomly selected 316 SNPs and carried out at least three replicates of direct PCR and Sanger sequencing to compare genotypes called from the resequencing data to evaluate the SNP accuracy rate (Tables S4 and S5). (2) We checked the previously published cotton SNP SLAF data (Su *et al*., 2016a) in 43 accessions with 20 206 SNPs (Table S6).

### Population structure

To build a phylogenetic tree, we selected a total of 10 180 SNPs at 4D SNPs and filtered all accessions with a MAF> 5% and missing rate per site < 10%. An unrooted phylogenetic tree was constructed using SNPhylo (Lee *et al*., 2014) and visualized with the R package of ‘ggtree’ (Yu *et al*., 2017). LD was calculated for each subpopulation (Wild, ESM and MLM) using SNPs with a MAF> 5% and missing rate per site < 10% using PLINK (Purcell *et al*., 2007). Each chromosome was separately calculated with the following parameters: (‐‐*r* ^2^ ‐‐ld‐window‐*r* ^2^ 0 ‐ld‐window‐kb 1000 ‐‐ld‐window 99999). The LD heat maps with surrounding peaks in the GWAS results were visualized using the R package of ‘LDheatmap’ (Shin *et al*., 2006). Using the same data set as LD, PCA was performed using GCAT (Yang *et al*., 2011a), and two‐dimensional coordinates were plotted for the 436 cotton accessions in R (www.r‐project.org) and the population structure was analysed with the fastSTRUCTURE (Raj *et al*., 2014) programme.

### Population genetic analysis and identification of selective sweeps

*F*_ST_ provides insights into the biology of evolutionary processes as a measure of population differentiation in genetic distance. To determine the pairwise *F*_ST_ values in two subpopulations, VCFtools (Danecek *et al*., 2011) software was used with a step size of 20 kb and a 100‐kb sliding window. The *π* value was used to measure each 20‐kb interval across the genome with a 100‐kb sliding window. In addition, we also employed SweepFinder2 to detect selective sweeps using the CLR statistic (DeGiorgio *et al*., 2016), and windows with the highest 5% of values and windows with a distance of ≤ 50 kb were merged into a single selected region.

### Bulk segregant analysis of the F_2_ population by whole‐genome resequencing

Two upland cotton accessions, ‘Zhong213’ and ‘Richmond6’, were used as parental lines to develop segregating populations for FT. Richmond6, a wild upland cotton with an FT of more than 100 days, was considered a line with extremely late flowering, and ‘Zhong213’, an excellent early maturing cultivar with an FT of approximately 65 days, was used as an early flowering cultivar (Li *et al*., 2017). In 2015, ‘Zhong213’ (female parent, P1) was crossed with Richmond6 (pollen donor, P2) in Sanya, Hainan, China (18°29′N, 109°52′E), to create F_1_ seeds from a single crossed plant. Then, the multiple F_1_ plants were planted during the winter and self‐pollinated to generate 500 F_2_ individuals in the same field. The F_2_ seeds were planted at the Cotton Research Institute of the Chinese Academy of Agricultural Sciences (CRICAAS), Anyang, Henan, China (36°08′N, 114°48′E), in 2017. Genomic DNA was isolated from young cotton leaves using the CATB method (Paterson *et al*., 1993). Bulked DNA samples were prepared by mixing an equal ratio from 30 individuals showing extremely early flowering and late flowering for sequencing on an Illumina HiSeq 4000 sequencer. A total of 278.61 Gb of sequence for 20 × in each parent and 30 × in each bulk sample was generated. Detailed information on the resequencing of parental lines and bulks is listed in Table S11. Short reads were aligned to the cotton reference genome (Wang *et al*., 2019) using BWA software (Li and Durbin, 2009), and SNP calling was performed using SAMtools (Li *et al*., 2009). SNPs with a base quality value >30 and read depth >10× were used for further analysis. The SNP index was calculated for all SNP positions using the method of Takagi *et al*. (2013). An average SNP index was calculated using a 1‐Mb sliding window and a step size of 10 kb, and a 95% confidence interval was used to obtain the average SNP index according to Lin *et al*. (2014).

### Phenotyping and genome‐wide association study

A total of 436 cotton accessions were selected for this study. For phenotyping, 355 accessions were planted at Anyang, Henan, China (36°08′N, 114°48′E), Shihezi, Xinjiang, China (44°31’N, 86°01′E) and Huanggang, Hubei, China (30°57′N, 114°92′E). From 2015 to 2017, multiple environmental evaluations were conducted in the three different cotton planting areas (YRR: Yellow River region, the YZRR: Yangtze River region, NIR: Northwest Inland region) throughout China (Table S1). Seven early maturity‐related traits were investigated in this study, including FT (days), FBP (days), WGP (days), YPBF (%), NFFB, HNFFB (cm) and PH (cm), as described previously (Jia *et al*., 2016; Li *et al*., 2017; Su *et al*., 2016b). The values of the best linear unbiased prediction (BLUP) of seven early maturity‐related traits in the 355 accessions were calculated using the R package of ‘lme4’ (Bates et al., 2014). A total 4 521 969 high‐quality SNPs (MAF > 0.05 and missing rate per site < 10%) were used to perform the GWAS for early maturity‐related traits in 355 accessions. A linear mixed model was used for marker association analysis with phenotype with GEMMA software (Zhou and Stephens, 2012). The Bonferroni‐corrected significance threshold was set as 1/*n* (*n* = total SNP number used in the association analysis).

### Gene expression analysis

qRT‐PCR: Total RNA was extracted using the Plant RNA Prep Pure Plant kit (Tiangen, Beijing, China). Then, cDNA was obtained using the SuperScript III First‐Stand Synthesis System (Takara, Dalian, China). Real‐time PCR was performed on an ABI 7500 Real‐Time PCR System (Foster City, CA). Gene expression levels were calculated using the 2^−ΔΔCT^ method (Livak and Schmittgen, 2001), and three biological replicates were used for each sample. The primers used for qRT‐PCR analysis are listed in Table S19.

### Transcriptome expression analysis

The flower bud was chosen to perform RNA‐seq at five developmental stages as follows: (1) square; (2) 5 days post‐square (5 DPS); (3) 10 DPS; (4) 15 DPS; (5) 20 DPS. Bracts were removed from each flower bud. Three biological replicates were collected for each stage. The libraries were constructed according to Zhu *et al*. (2018) and sequenced on the BGISEQ‐500 platform using 100‐bp paired‐end sequencing. The expression level of each gene was calculated using fragments per kilobase of exon model per million mapped reads (FPKM) determined by DESeq2 (Love *et al*., 2014) and visualized using custom R scripts. The RNA‐Seq data PRJNA248163 were obtained from NCBI (https://www.ncbi.nlm.nih.gov/bioproject/). The expression value of each gene was determined using GFOLD software (Feng *et al*., 2012). Differentially expressed genes were defined at *P* < 0.05 using Student’s *t*‐test, expression changes of at least twofold, and expression levels of at least 1 FPKM in at least one stage during ovule and fibre development.

### Virus‐induced gene silencing in cotton

Approximately 300 bp of the gene‐specific region for *Ghir_D03G011310* and *GhGA2OX8* was inserted into the pCLCrVA vector and pCLCrVB as the helper vector (Gu *et al*., 2014). pCLCrVA::*Ghir_D03G011310*, pCLCrVA::*GhGA2OX8* and pCLCrVB were transferred into cotton cotyledons of ‘CRI50’ through *Agrobacterium tumefaciens* strain LBA4404 as previously described (Gu *et al*., 2014). The primers used for construction of the VIGS vector are listed in Table S19.

### Genetic transformation of *Arabidopsis thaliana*

The full‐length open reading frame of *GhGA2OX8* was amplified by PCR using cDNAs synthesized from RNA that was isolated from ‘CRI50’ corresponding to the SNP allele. The amplified products were further inserted into the binary expression vector pBI121 driven by the 35S promoter to generate the *35S::GhGA2OX8* construct. The resulting construct was individually introduced into *Agrobacterium tumefaciens* strain GV3101 and transformed into the *Arabidopsis* ecotype Columbia. The homozygous T3 generation was further verified by PCR. The primers used for gene cloning are listed in Table S19.

### Determination of endogenous GAs

‘CRI50’ and ‘TM‐1’ were grown under greenhouse conditions for approximately 6 weeks. To analyse GA content, the apical buds of ‘CRI50’ and ‘TM‐1’ were collected at the three leaf stage and four leaf stage, and they were analysed using both high‐performance liquid chromatography fluorescence and LC‐MS/MS according to Li *et al*. (2011). Each sample was mixed in equal amounts, and three biological and three technical replicates were evaluated.

### Tissue sectioning analysis

For paraffin section observation, elongated mature tissue of the first internode was cut from ‘CRI50’, CLCrVA and VIGS lines and placed in formalin‐acetic acid‐alcohol (FAA) mixed solution for no less than 48 h, and then the samples were dehydrated with a graded ethanol series. The prepared sections were sequentially stained, infiltrated with xylene and finally mounted beneath a coverslip. The sections were then dewaxed twice in dimethylbenzene and each time for 10 min. Finally, light microscopy was performed using microscope and photographed.

## Funding

This research was supported by the National Key Research and Development Program of China (2016YFD010401, 2016YED0101006), the China Agriculture Research System (No. CARS‐18) and the National Natural Science Foundation of China (31601346, 31621005).

## Competing interests

The authors declare no competing interests.

## Authors’ contributions

SY, JH and LL designed the experiments; LL and CZ performed most of the experiments; GL and LG provided technical assistance; LL and QL performed data analysis; HW and HW revised the language. SY and LL supervised and complemented the writing. All authors read and approved the final manuscript.

## Availability of supporting data and materials

The raw data for resequencing, RNA‐seq and Sanger sequencing results have been deposited in the NCBI database under PRJNA389777, PRJNA431876, PRJNA509318 and MK847254‐MK844855. The accession numbers are summarized in Table S1. Phenotype information used in GWAS is summarized in Table S20.

## Supporting information


**Table S1** Summary of the 436 accessions for resequencing.
**Table S2** Summary of SNPs in each chromosome.
**Table S3** Summary of Indels in each chromosome.
**Table S4** Summary of accessions used in PCR and Sanger sequencing.
**Table S5** SNP loci selected for validation PCR and Sanger sequencing.
**Table S6** SNP loci selected for validation by the SLAF SNP dataset.
**Table S7** Putative regions identified to be under domestication selection sweeps.
**Table S8** Genes within the putative domestication sweeps.
**Table S9** Candidate genes involved in significantly enriched biological process GO terms, including flowering time, hormone catabolism, defence responses and ageing, in selection sweeps.
**Table S10** Summary of known early maturity‐related QTLs during domestication.
**Table S11** Resequencing information of parental lines and bulks for BSA analysis.
**Table S12** ANNOVA and a broad sense heritability of early maturity‐related traits in the 355 accessions.
**Table S13** Genome‐wide significant association signals for seven early maturity‐related traits in 355 accessions using the LMM method.
**Table S14** 237 SNPs in the GWAS peaks on the chromosome A05.
**Table S15** GWAS peaks haplotype on chromosome A05 and D03.
**Table S16** Comparison of the plant height for *GhGA2OX8*‐transgenic *Arabidopsis*.
**Table S17** Elite loci associated with yield and fibre quality from previous studies used in the present study.
**Table S18** Differentially expressed genes during fibre and ovule development.
**Table S19** List of all primers used in this study.
**Table S20** Seven early maturity‐related traits used for GWAS

**Figure S1** Map of the early maturity cotton growing area and early maturity cotton used in this study, including the cotton growing area of the Yellow River region (YRR), north of the Northwest Inland region (NIR), and the Northern Specific Early Maturity region (NSER).
**Figure S2** Circos plot showing SNP diversity across the 26 chromosomes of *Gossypium hirsutum*. The chromosomes are numbered. The blue circle represents SNP density; the purple circle shows Indel diversity; the orange and green colours represent gene density within SNP and Indel markers, respectively.
**Figure S3** The SNP number on each chromosome and distributions at different regions.
**Figure S4** The Indel number on each chromosome and distributions at different regions.
**Figure S5** Morphological changes in early, medium and late maturity cotton and wild cotton during domestication and improvement. a: cotton at the stage when the node of the first fruiting branch flower is opening in early maturity cotton. b: cotton at the stage when all the balls are open in early maturity cotton.
**Figure S6** Model‐based clustering analysis with different numbers of clusters (*K* = 2 and 3).
**Figure S7** Nucleotide diversity (π) and population divergence (*F*_ST_) across the three population, and a diagram of early maturity and special early maturity (ESM) population and medium and late maturity (MLM) population.
**Figure S8** Decay of linkage disequilibrium (LD) in Wild, early maturity and special early maturity (ESM) population and medium and late maturity (MLM) population.
**Figure S9** Pedigree information for early maturity cotton breeding. The accessions in green are the founder cultivars of early maturity cotton; the accessions in red were collected and analysed in our study; the accessions in black were early maturity cotton but not analysed in our study.
**Figure S10** The frequency of FT (flowering time) among the parents (‘Zhong213’ and ‘Richmondi 6’) and 500 F2 individuals in Anyang 2017.
**Figure S11** Distributions of the mean values of seven early maturity‐related traits of 355 accessions in eight environments, including flowering time (FT), the period from first flower blooming to first boll opening (FBP), whole growth period (WGP), yield percentage before frost (YPBF), node of the first fruiting branch (NFFB), height of the node of the first fruiting branch (HNFFB) and plant height (PH).
**Figure S12** Correlation analysis of seven early maturity traits in 355 natural populations, including flowering time (FT), the period from first flower blooming to first boll opening (FBP), whole growth period (WGP), yield percentage before frost (YPBF), node of the first fruiting branch (NFFB), height of the node of the first fruiting branch (HNFFB) and plant height (PH). **Indicates significance at 0.01.
**Figure S13** Manhattan plots for BLUP of flowering time (FT), the period from first flower blooming to first boll opening (FBP), whole growth period (WGP), yield percentage before frost (YPBF), node of the first fruiting branch (NFFB), height of the node of the first fruiting branch (HNFFB) and plant height (PH). Significant trait‐associated SNPs are distinguished by red lines.
**Figure S14** Manhattan plots for flowering time (FT) in separate environment.
**Figure S15** Manhattan plots for the period from first flower blooming to first boll opening (FBP) in separate environment.
**Figure S16** Manhattan plots for whole growth period (WGP) in separate environment.
**Figure S17** Manhattan plots for yield percentage before frost (YPBF) in separate environment.
**Figure S18** Manhattan plots for node of the first fruiting branch (NFFB) in separate environment.
**Figure S19** Manhattan plots for height of the node of the first fruiting branch (HNFFB) in separate environment.
**Figure S20** Manhattan plots for plant height (PH) in separate environment.
**Figure S21** Expression profiles of Ghir_D03G011290 and Ghir_D03G011300. The *x*‐axis represents developmental stages (0, 5, 10, 15 and 20 DPS), and the y‐axis indicates the relative expression levels as determined by RNA‐seq. The error bars indicate standard deviation of three biological replicates.
**Figure S22** Expression levels of Ghir_D03G011310 at three leaf growth stages and four leaf growth stages by qRT‐PCR (**indicates significance at the 0.01 probability level).
**Figure S23** Gene structure of Ghir_D03G011310 and the polymorphism in two haplotypes, ‘A’ allele and ‘G’ allele.
**Figure S24** (a) HRM analysis for SNP (rsD03_39122594) in recombinant inbred line population. The axis of the outside is original melting curves; the axis of the inside is melting curves after logarithm. Red and blue curves correspond to favourable alleles (A) and unfavourable alleles (G), respectively. (b) Box plots for two haplotypes in whole growth period at recombinant inbred line population mentioned above (** *P* < 0.01).
**Figure S25** Expression level of Ghir_D03G011310 in empty control as CK and VIGS plants. **Indicates significance at 0.01.
**Figure S26** Nucleotide diversity and population divergence (FST) on the chromosome D03 (red part is the strong LD block regions). (a) Nucleotide diversity across the three population. (b) Population divergence (FST) between early maturity and special early maturity (ESM) population and medium and late maturity (MLM) population.
**Figure S27** Box plots for SNP rsA05_16453277 (G/T) in height of the node of the first fruiting branch (HNFFB) and plant height (PH).
**Figure S28** Comparison of Ghir_A05G017290 expression levels between ‘CRI50’ (green) and ‘TM‐1’ (red) during developmental stages (0, 5, 10, 15, and 20 DPS) by RNA‐seq. **Indicates significance at 0.01.
**Figure S29** Manhattan plots for HNFFB on chromosome D05.
**Figure S30** Morphological phenotypes of wild‐type and Arabidopsis containing the 35S::GhGA2OX8 cDNA construct. WT, OE8 and OE9 represent wild‐type and transgenic lines, respectively.
**Figure S31** Comparison of cell length of CRI50, CLCrVA and VIGS lines. **Indicates significance at 0.01.
