# Supplementary material for: Genomic analyses reveal the genetic basis of early maturity and identification of loci and candidate genes in upland cotton (Gossypium hirsutum L.)
Source: Plant Biotechnol J. 2020 Aug 1;19(1):109–23. doi: 10.1111/pbi.13446 (PMC7769233; doi:10.1111/pbi.13446)
Supplement: Supplementary file 2 — Figure S1 Map of the early maturity cotton growing area and early maturity cotton used in this study, including the cotton growing area of the Yellow River region (YRR), north of the Northwest Inland region (NIR), and the Northern Specific Early Maturity region (NSER). Figure S2 Circos plot showing SNP diversity across the 26 chromosomes of Gossypium hirsutum. The chromosomes are numbered. The blue circle represents SNP density; the purple circle shows Indel diversity; the orange and green colours represent gene density within SNP and Indel markers, respectively. Figure S3 The SNP number on each chromosome and distributions at different regions. Figure S4 The Indel number on each chromosome and distributions at different regions. Figure S5 Morphological changes in early, medium and late maturity cotton and wild cotton during domestication and improvement. a: cotton at the stage when the node of the first fruiting branch flower is opening in early maturity cotton. b: cotton at the stage when all the balls are open in early maturity cotton. Figure S6 Model‐based clustering analysis with different numbers of clusters (K = 2 and 3). Figure S7 Nucleotide diversity (π) and population divergence (F ST) across the three population, and a diagram of early maturity and special early maturity (ESM) population and medium and late maturity (MLM) population. Figure S8 Decay of linkage disequilibrium (LD) in Wild, early maturity and special early maturity (ESM) population and medium and late maturity (MLM) population. Figure S9 Pedigree information for early maturity cotton breeding. The accessions in green are the founder cultivars of early maturity cotton; the accessions in red were collected and analysed in our study; the accessions in black were early maturity cotton but not analysed in our study. Figure S10 The frequency of FT (flowering time) among the parents (‘Zhong213’ and ‘Richmondi 6’) and 500 F2 individuals in Anyang 2017. Figure S11 Distributions of the mean values of [file PBI-19-109-s002.pdf]

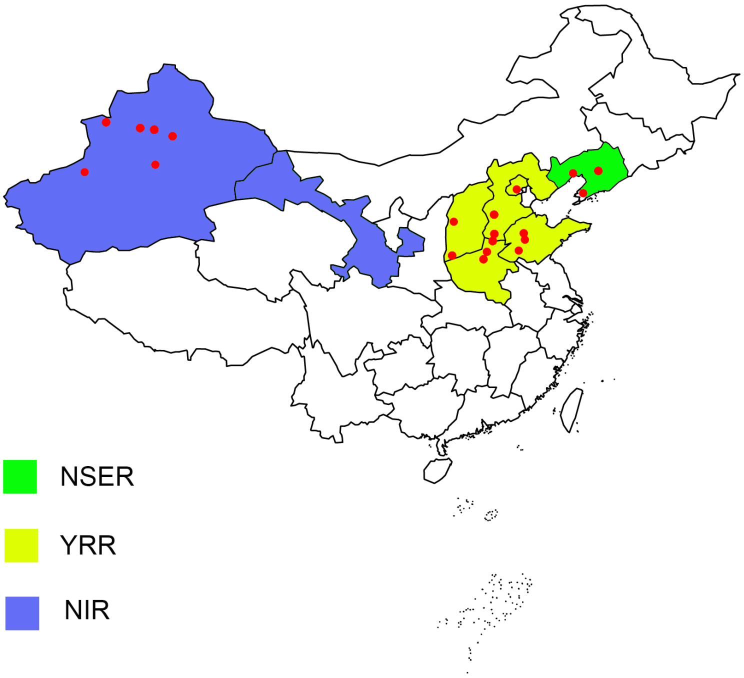

Figure S1: Map of the early maturity cotton growing area and early maturity cotton used in this study.

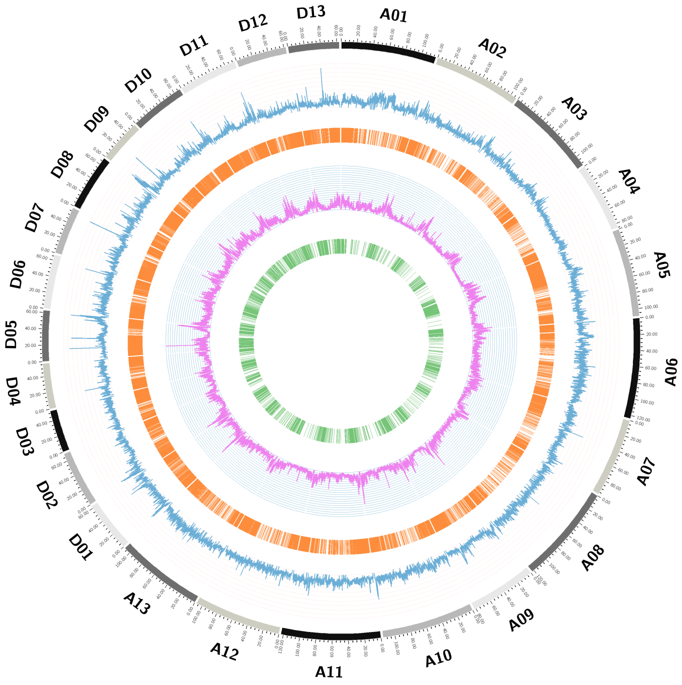

Figure S2: Circos plot showing SNP diversity across the 26 chromosomes of *Gossypium hirsutum*. The chromosomes are numbered. The blue circle represents SNP density; the red circle shows Indel diversity; the orange and green colors represent gene density within SNP and Indel markers, respectively.

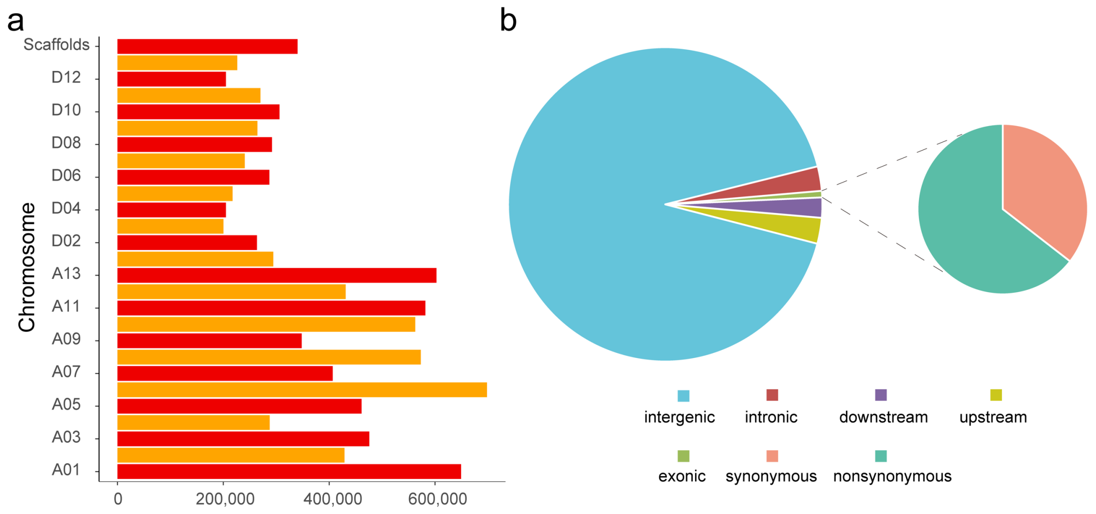

Figure S3: The SNP number on each chromosome and distributions at different regions.

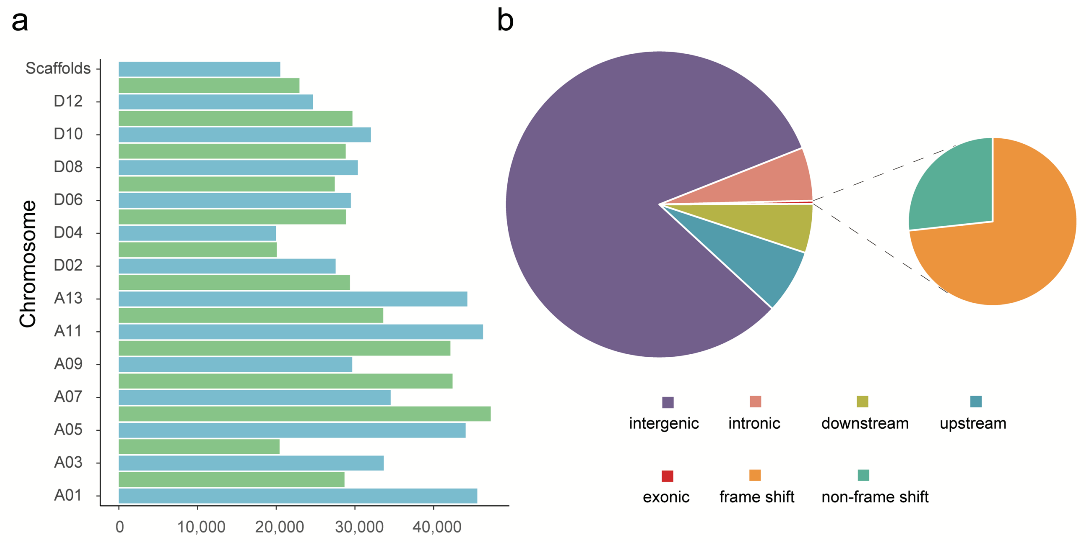

Figure S4: The Indel number on each chromosome and distributions at different regions.

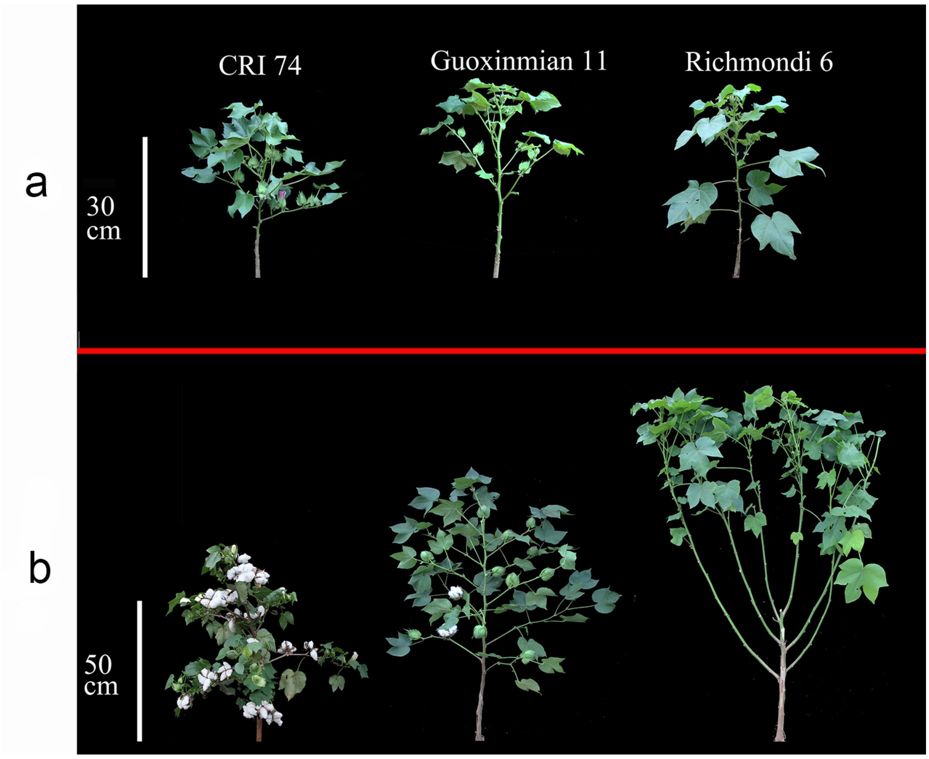

Figure S5: Morphological changes in early, medium and late maturity cotton and wild cotton during domestication and improvement. a: cotton at the stage when the node of the first fruiting branch flower is opening in early maturity cotton. b: cotton at the stage when all the bolls are open in early maturity cotton.

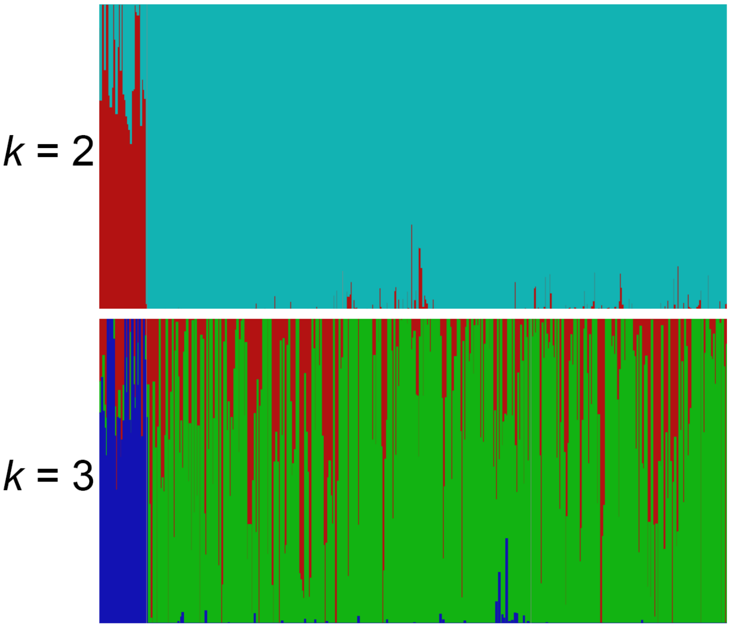

Figure S6: Model-based clustering analysis with different numbers of clusters (K = 2 and 3).

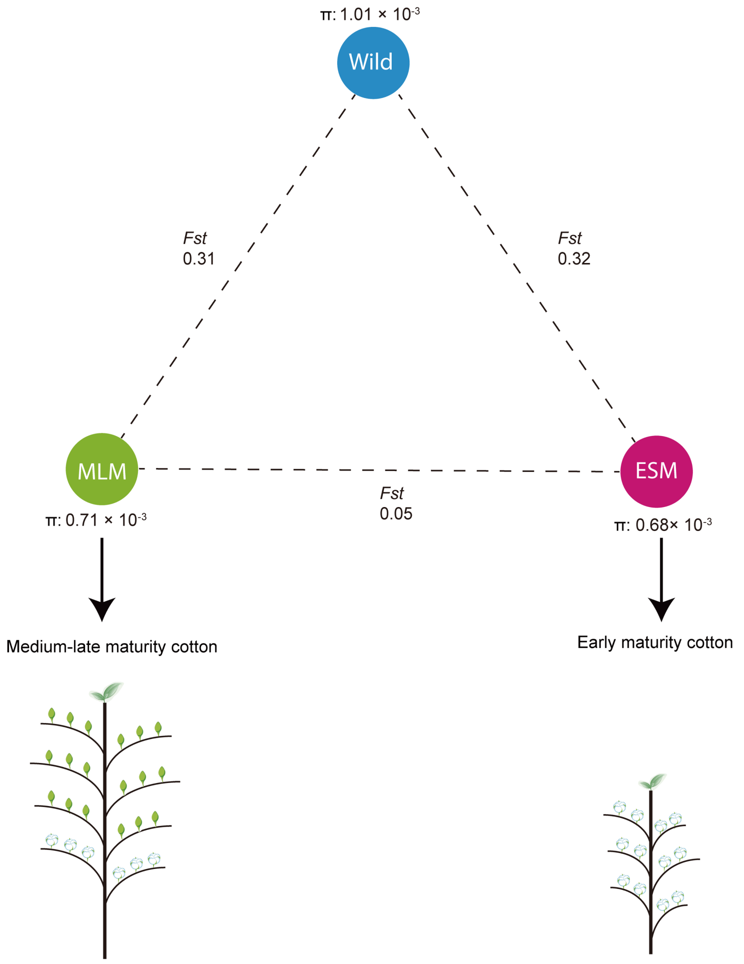

Figure S7: Nucleotide diversity (π) and population divergence (F_{ST}) across the three population, and a diagram of ESM and MLM.

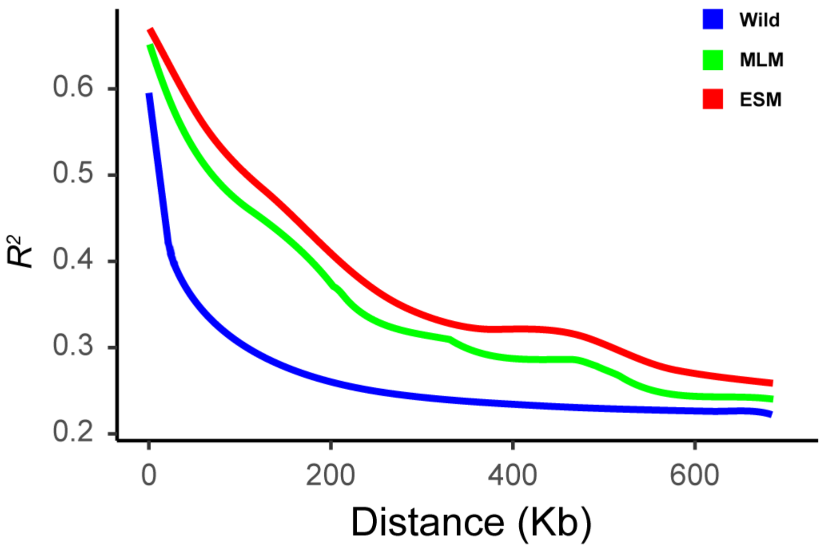

Figure S8: Decay of linkage disequilibrium (LD) in Wild, MLM and ESM.

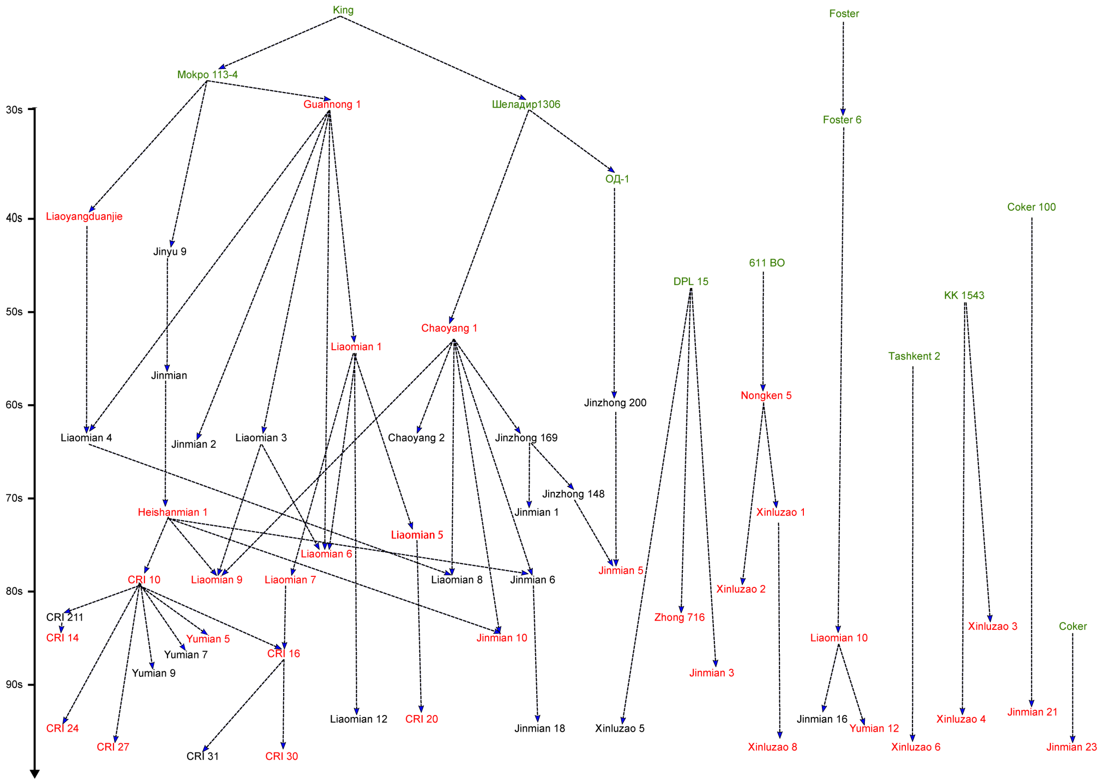

Figure S9: Pedigree information for early maturity cotton breeding. The accessions in green are the founder cultivars of early maturity cotton; the accessions in red were collected and analyzed in our study; the accessions in black were early maturity cotton but not analyzed in our study.

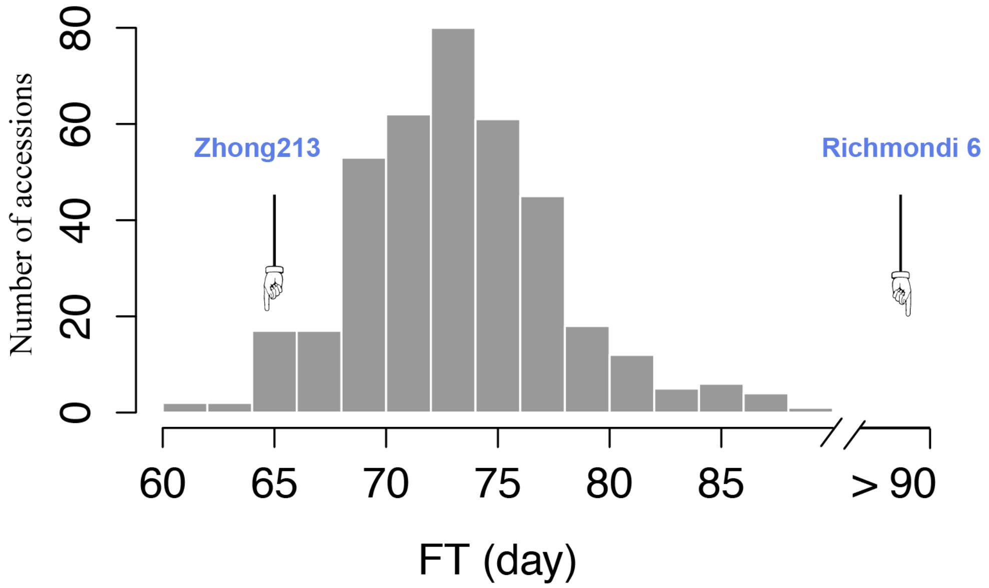

Figure S10: The frequency of FT (flowering time) among the parents (Zhong213 and Richmondi 6) and 500 F_2 individuals in Anyang 2017.

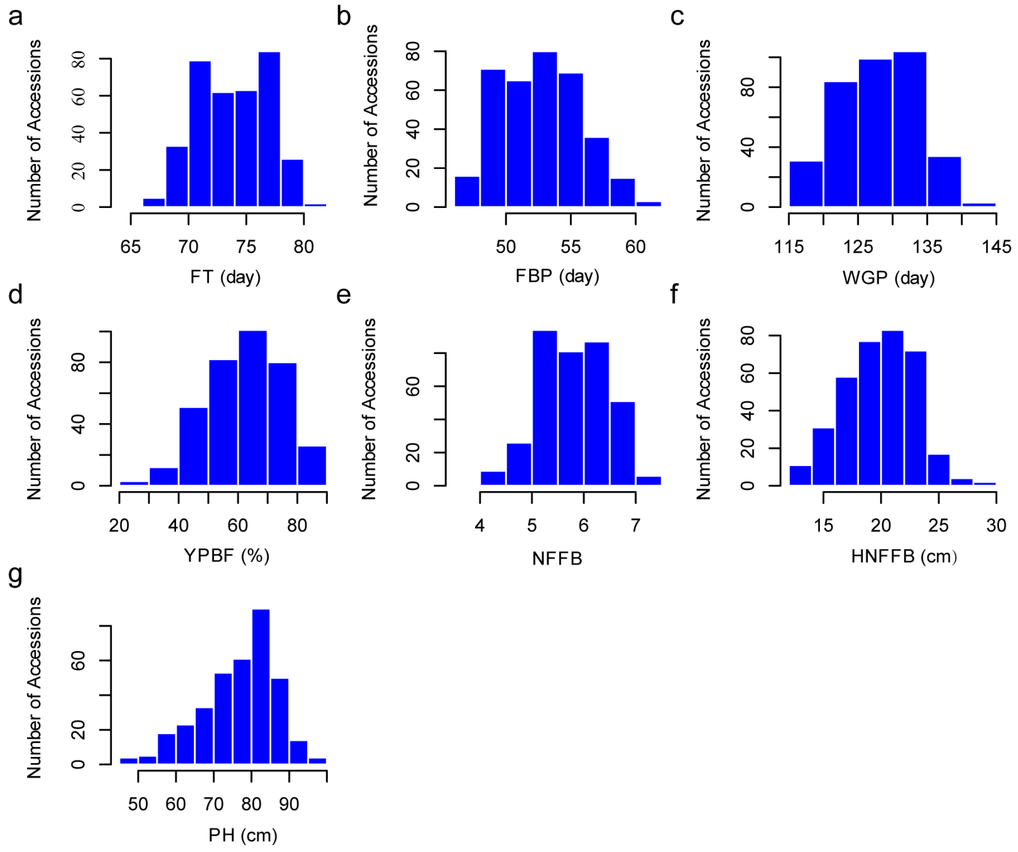

Figure S11: Distributions of the mean values of seven early maturity-related traits of 355 accessions in eight environments, including flowering time (FT), the period from first flower blooming to first boll opening (FBP), whole growth period (WGP), yield percentage before frost (YPBF), node of the first fruiting branch (NFFB), height of the node of the first fruiting branch (HNFFB), and plant height (PH).

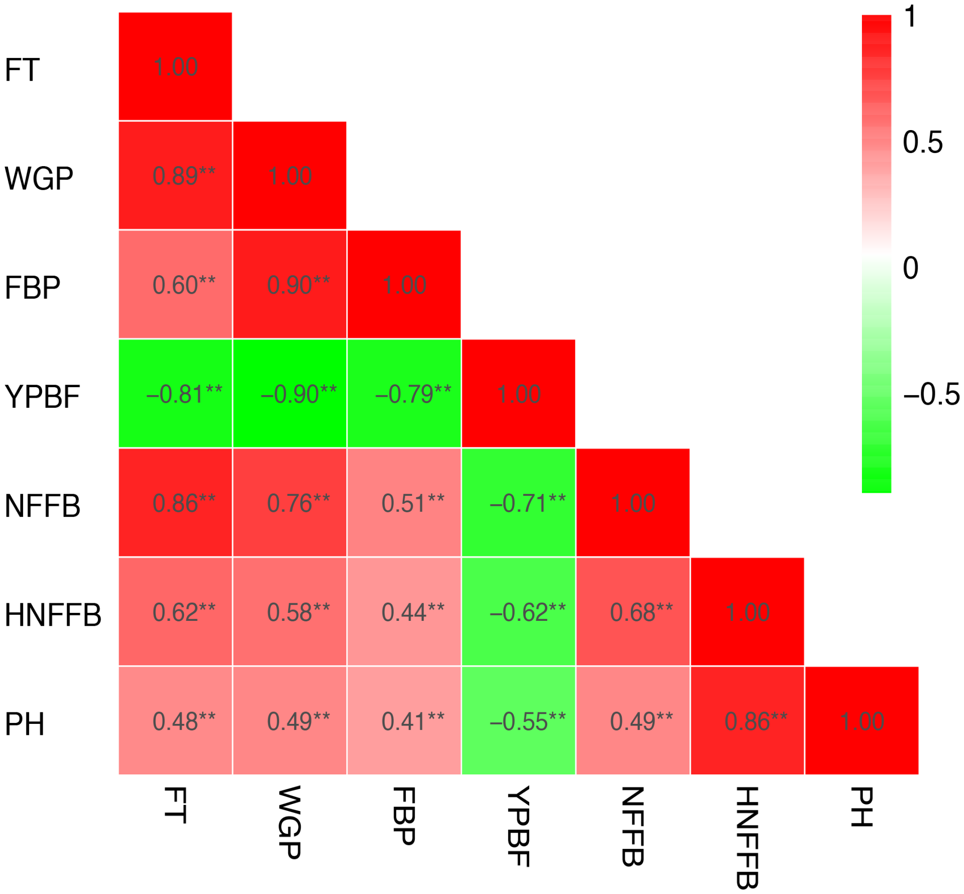

Figure S12: Correlation analysis of seven early maturity traits in 355 natural populations, including flowering time (FT), the period from first flower blooming to first boll opening (FBP), whole growth period (WGP), yield percentage before frost (YPBF), node of the first fruiting branch (NFFB), height of the node of the first fruiting branch (HNFFB), and plant height (PH).

**Indicates significance at 0.01.

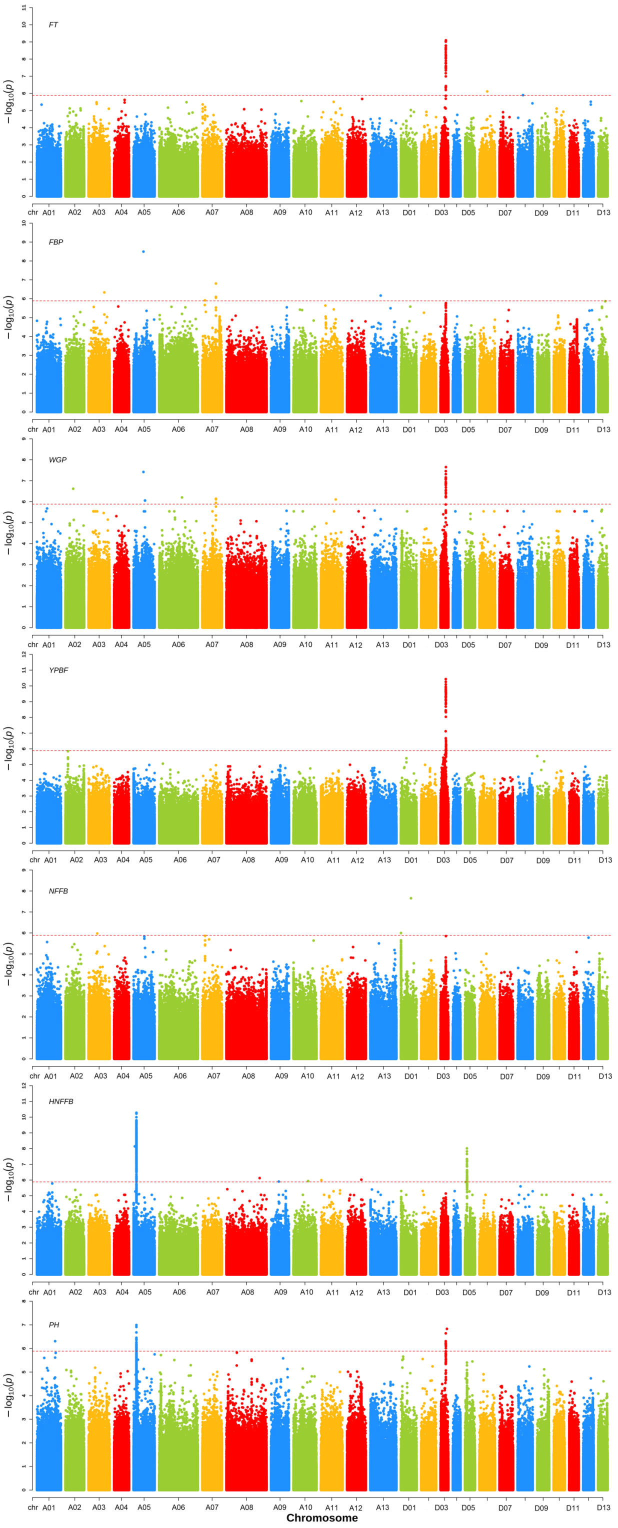

Figure S13: Manhattan plots for BLUP of flowering time (FT), the period from first flower blooming to first boll opening (FBP), whole growth period (WGP), yield percentage before frost (YPBF), node of the first fruiting branch (NFFB), height of the node of the first fruiting branch (HNFFB), and plant height (PH). Significant trait-associated SNPs are distinguished by red lines.

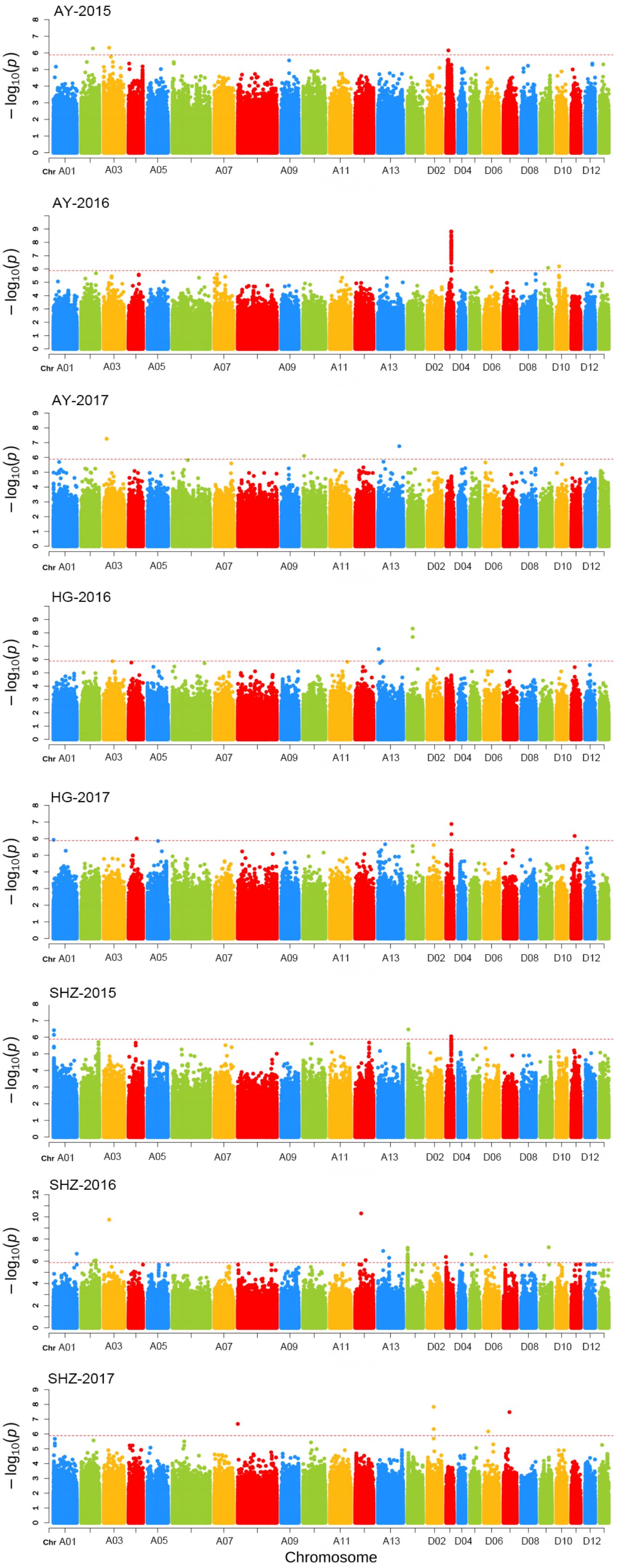

Figure S14: Manhattan plots for flowering time (FT) in separate environment.

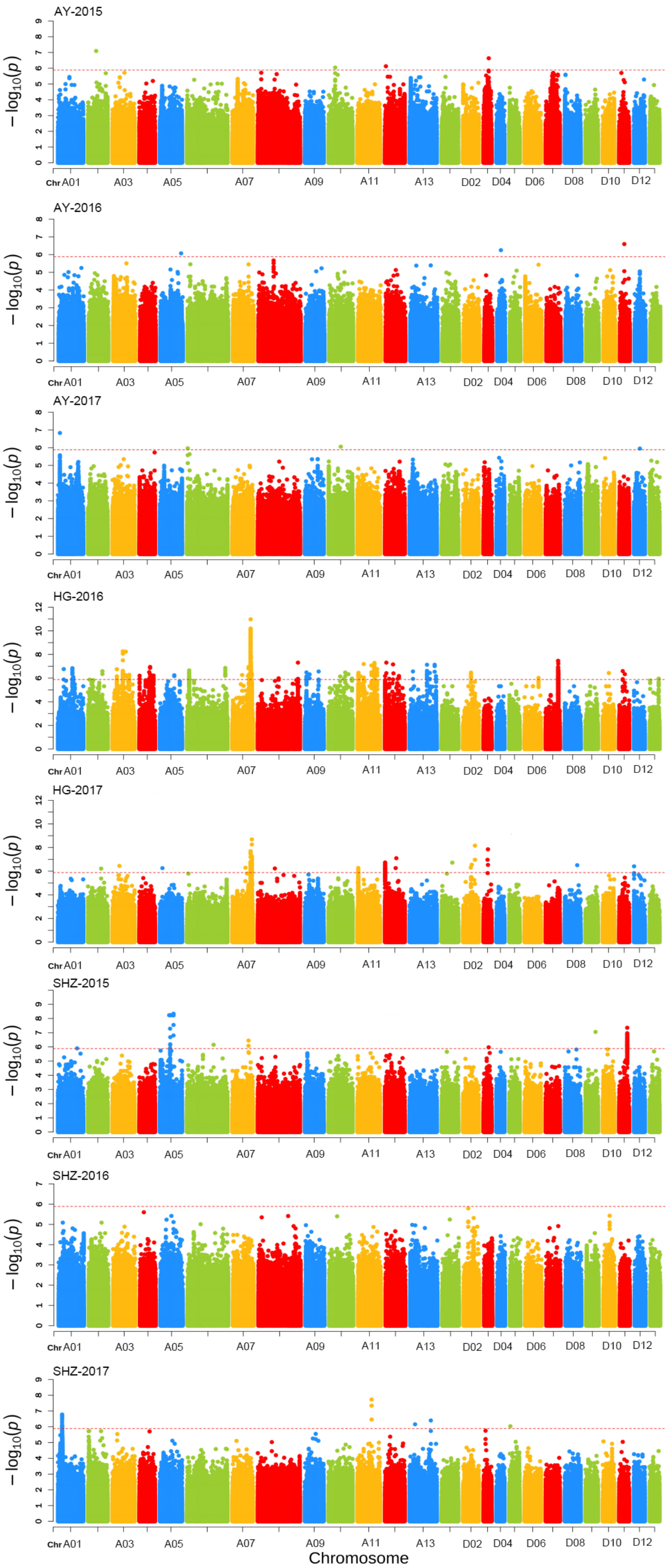

Figure S15: Manhattan plots for the period from first flower blooming to first boll opening (FBP) in separate environment.

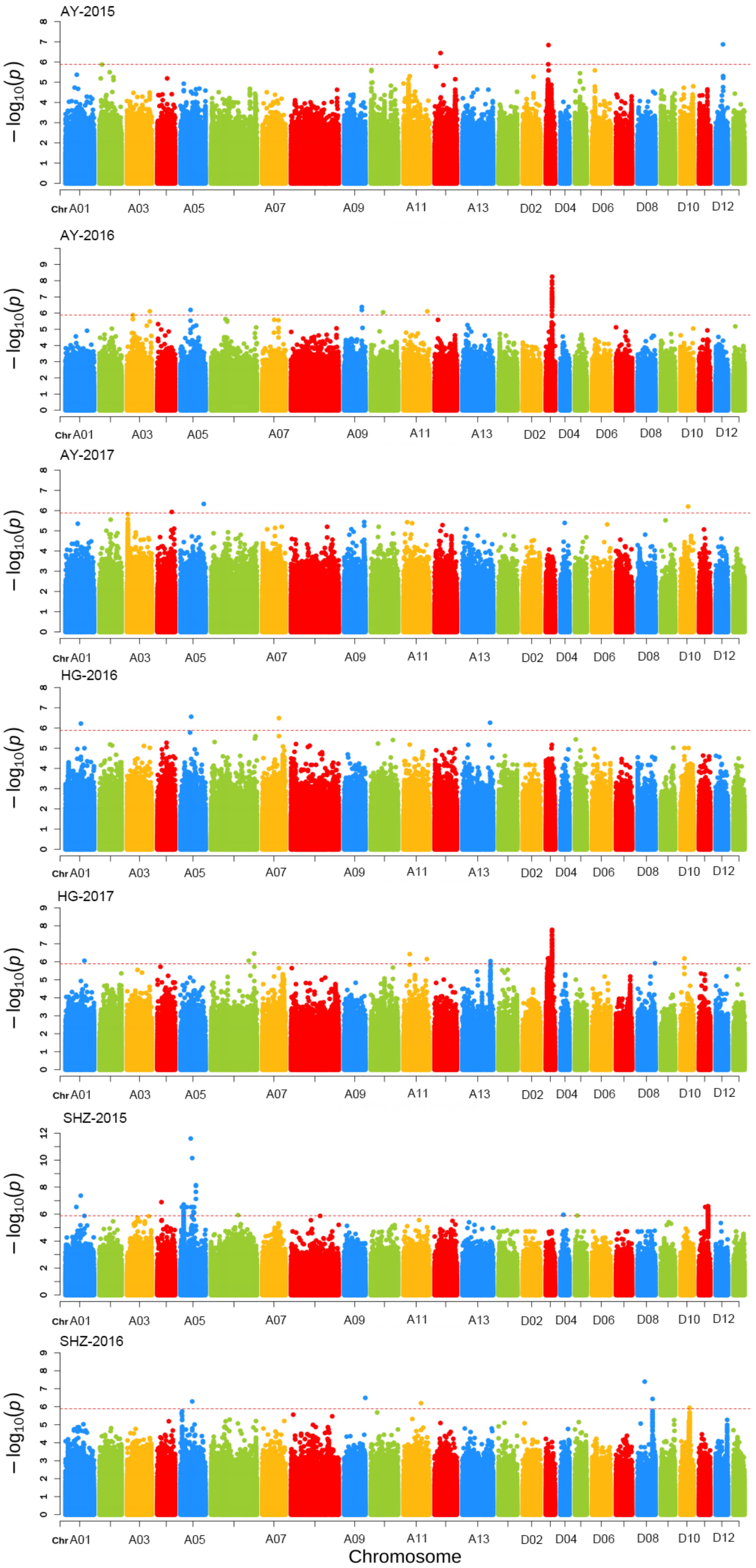

Figure S16: Manhattan plots for whole growth period (WGP) in separate environment.

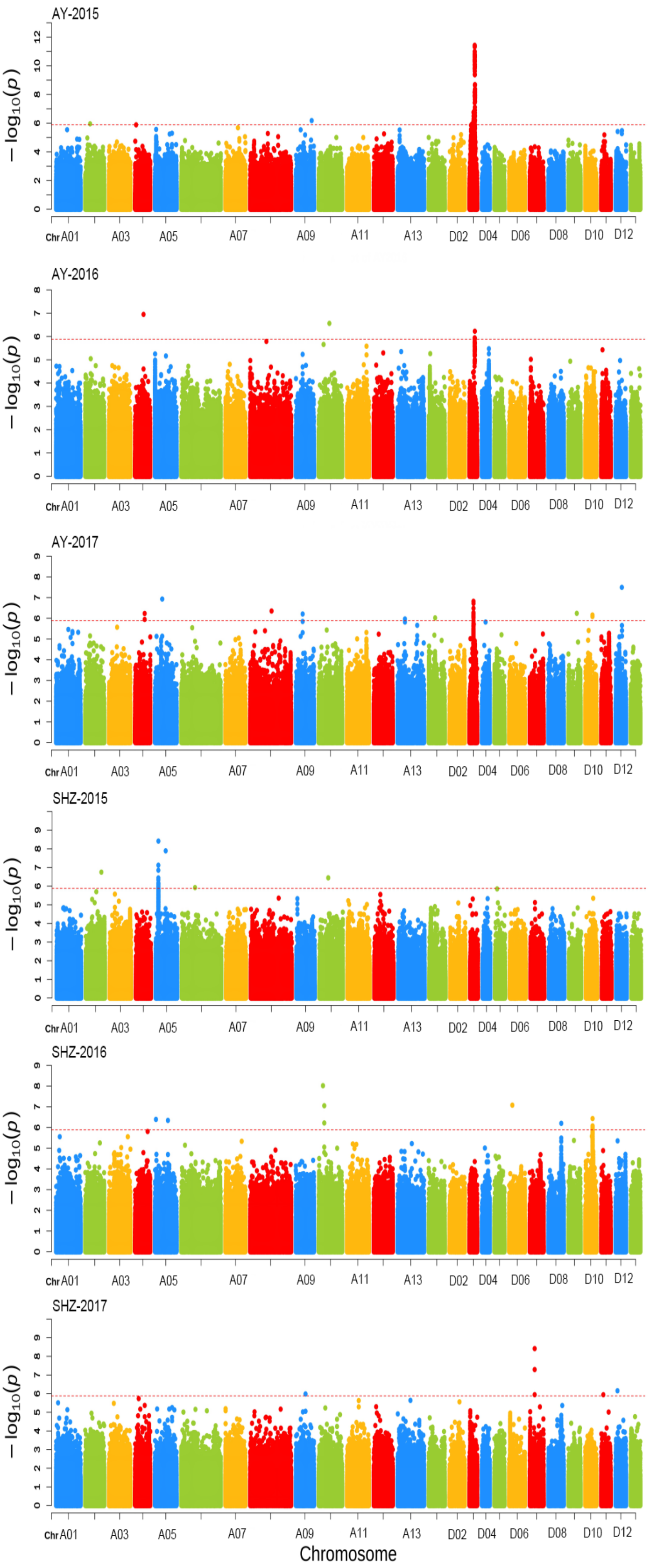

Figure S17: Manhattan plots for yield percentage before frost (YPBF) in separate environment.

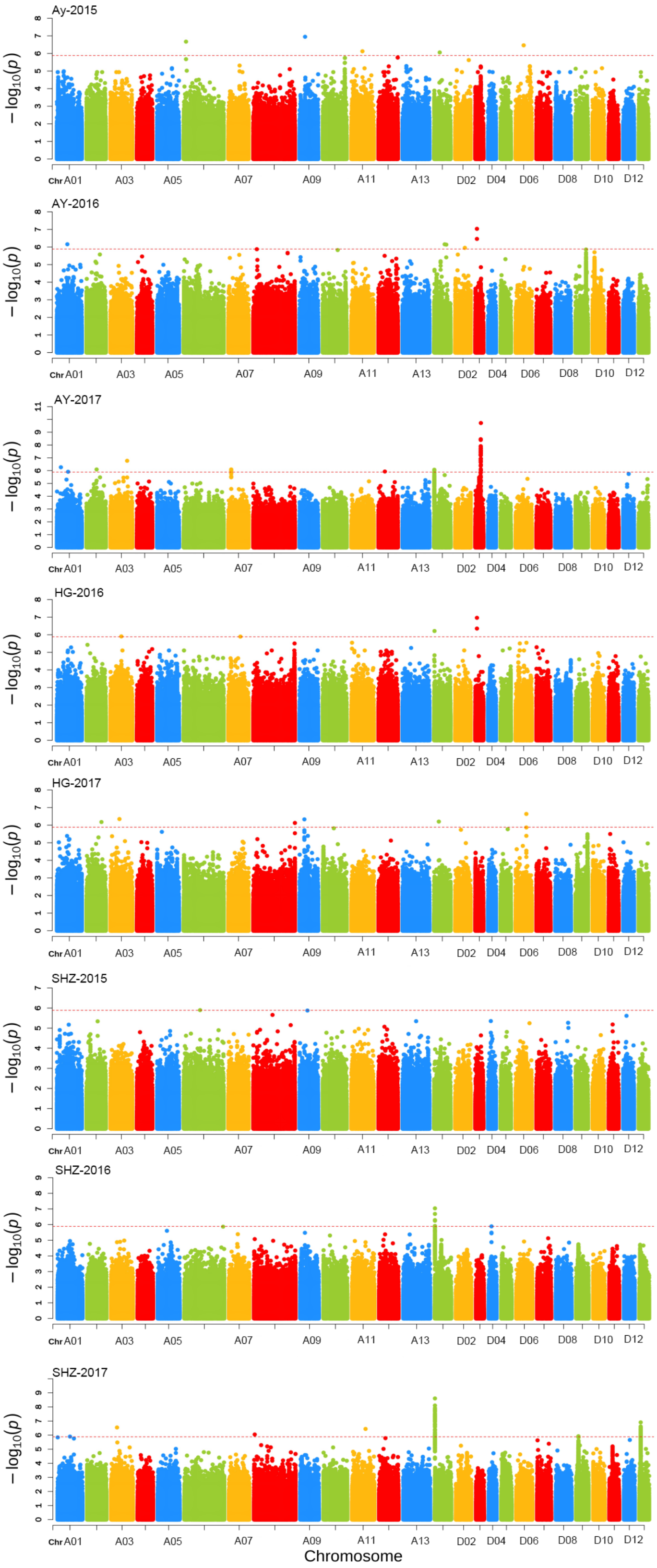

Figure S18 Manhattan plots for node of the first fruiting branch (NFFB) in separate environment.

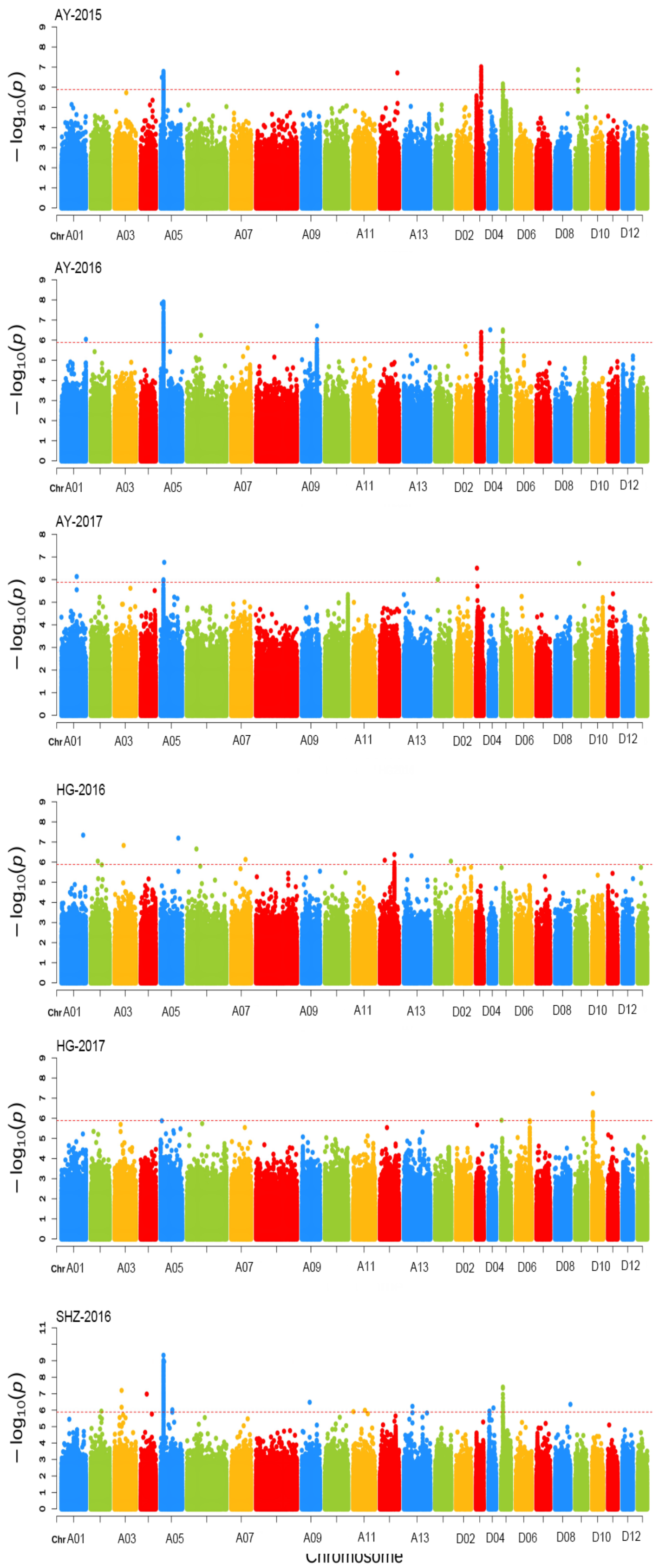

Figure S19: Manhattan plots for height of the node of the first fruiting branch (HNFFB) in separate environment.

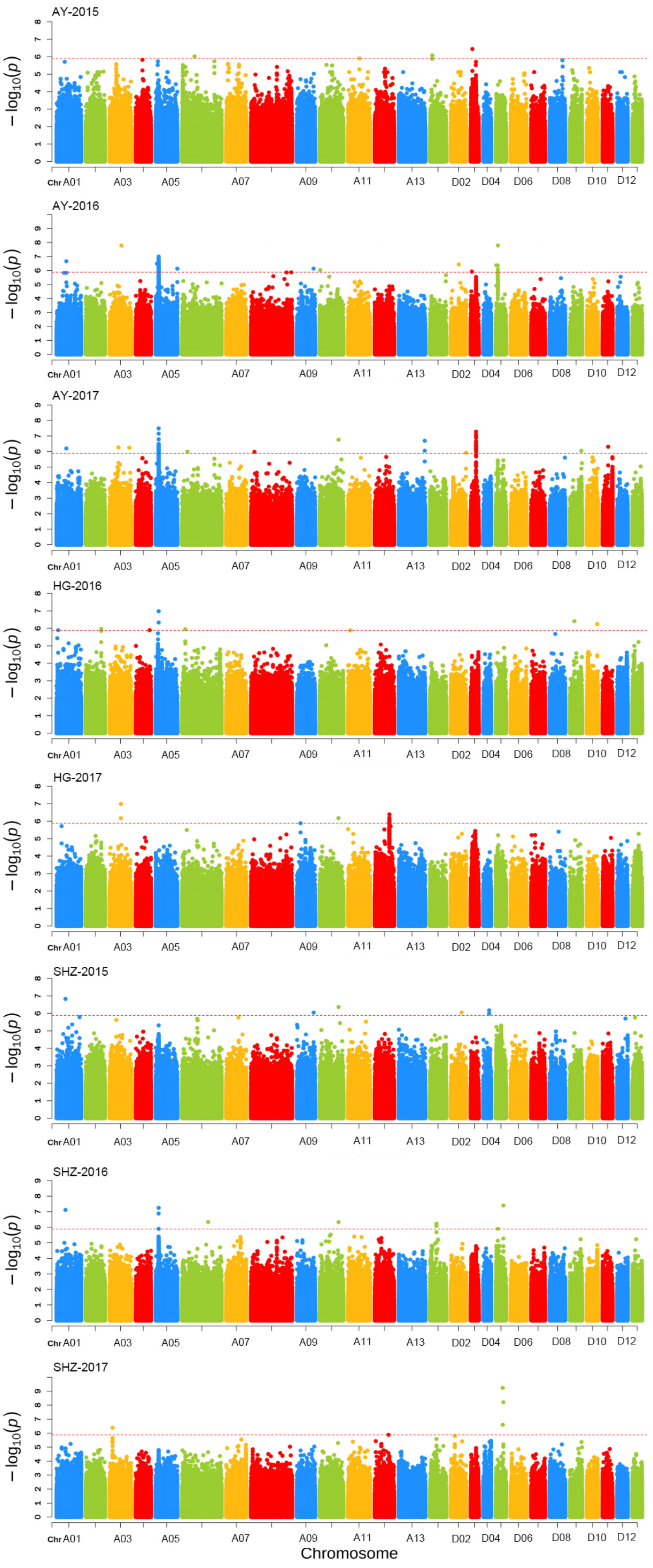

Figure S20: Manhattan plots for plant height (PH) in separate environment.

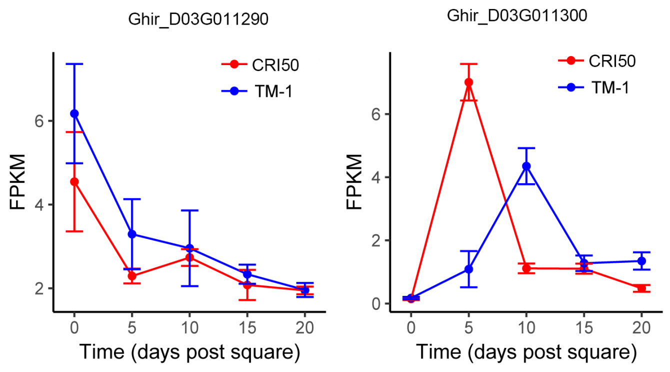

Figure S21: Expression profiles of *Ghir_D03G011290* and *Ghir_D03G011300*. The x-axis represents developmental stages (0, 5, 10, 15, and 20 DPS), and the y-axis indicates the relative expression levels as determined by RNA-seq. The error bars indicate standard deviation of three biological replicates.

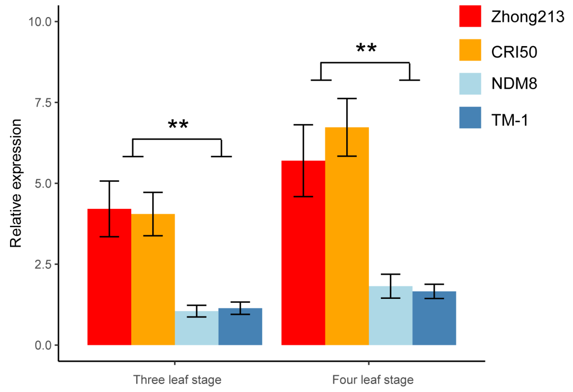

Figure S22: Expression levels of *Ghir_D03G011310* at three leaf growth stages and four leaf growth stages by qRT-PCR.
**indicates significance at the 0.01 probability level.

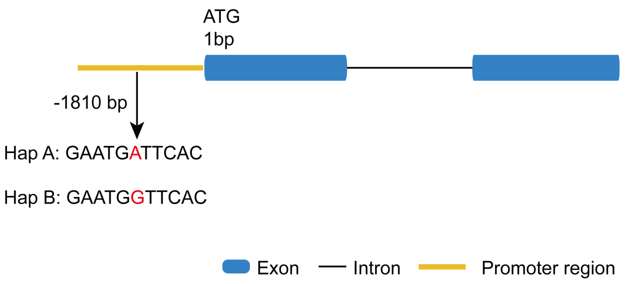

Figure S23: Gene structure of *Ghir_D03G011310* and the polymorphism in two haplotypes, ‘A’ allele and ‘G’ allele.

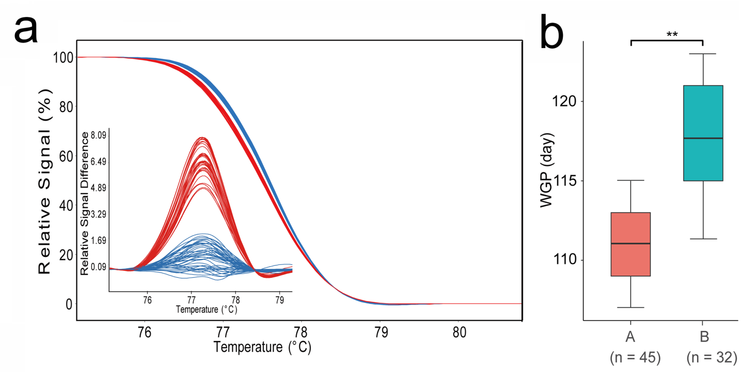

Figure S24: (a) HRM analysis for SNP (rsD03_39122594) in recombinant inbred line population. The axis of the outside is original melting curves; the axis of the inside is melting curves after logarithm. Red and blue curves correspond to favorable alleles (A) and unfavorable alleles (G), respectively. (b) Box plots for two haplotypes in whole-growth period at recombinant inbred line population mentioned above.

** P < 0.01.

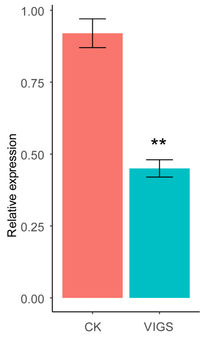

Figure S25: Expression level of *Ghir_D03G011310* in empty control as CK and VIGS plants.

**Indicates significance at 0.01.

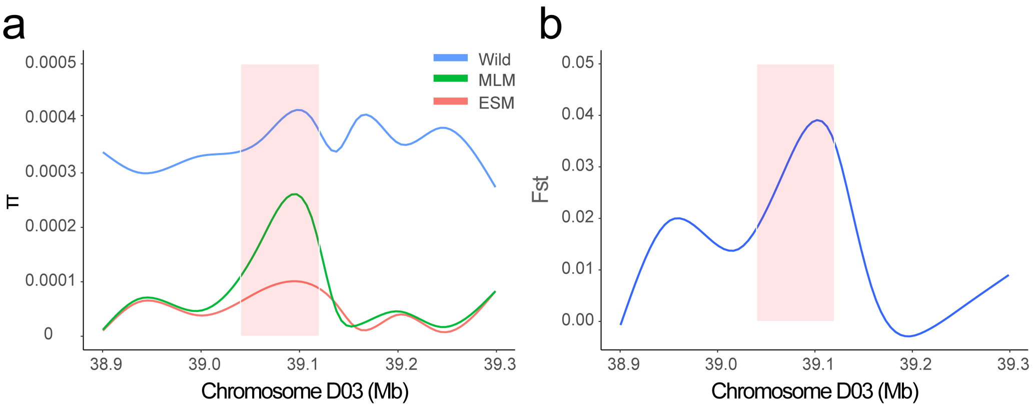

Figure S26: Nucleotide diversity and population divergence (F_{ST}) on the chromosome D03 (red part is the strong LD block regions). (a) Nucleotide diversity across the three population. (b) Population divergence (F_{ST}) between early maturity and special early maturity (ESM) population and medium and late maturity (MLM) population.

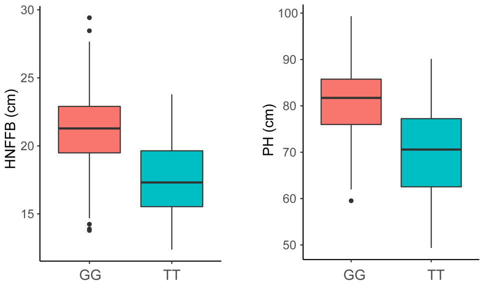

Figure S27: Box plots for SNP rsA05_16453277 (G/T) in height of the node of the first fruiting branch (HNFFB) and plant height (PH).

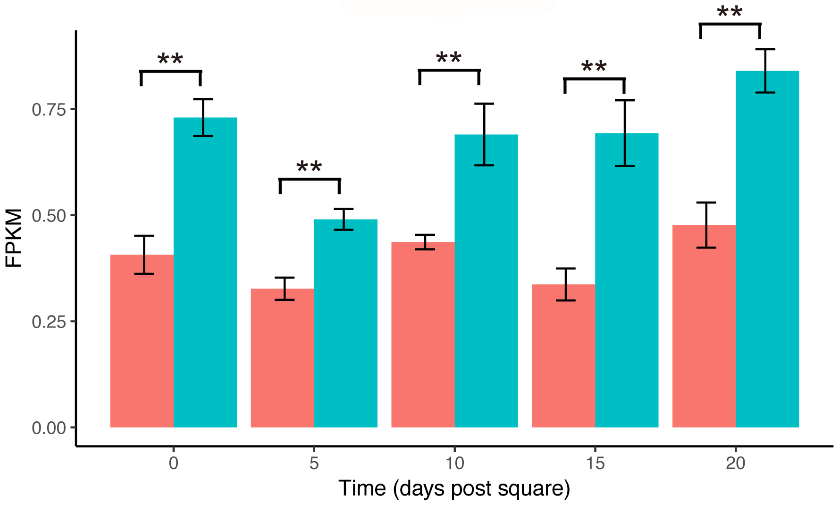

Figure S28: Comparison of *Ghir_A05G017290* expression levels between ‘CRI50’ (green) and ‘TM-1’ (red) during developmental stages (0, 5, 10, 15, and 20 DPS) by RNA-seq.

**Indicates significance at 0.01.

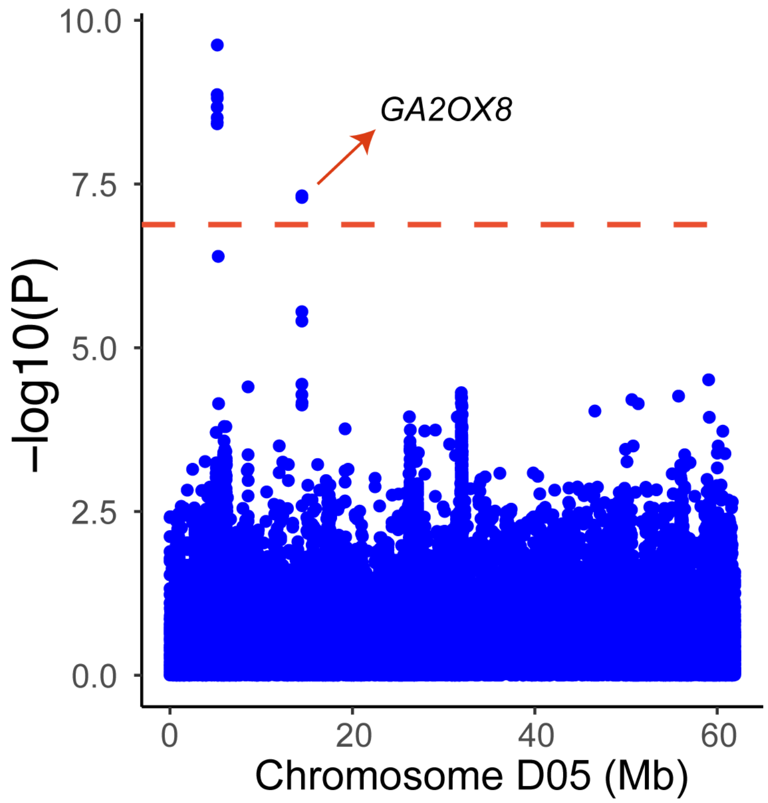

Figure S29: Manhattan plots for HNFFB on chromosome D05.

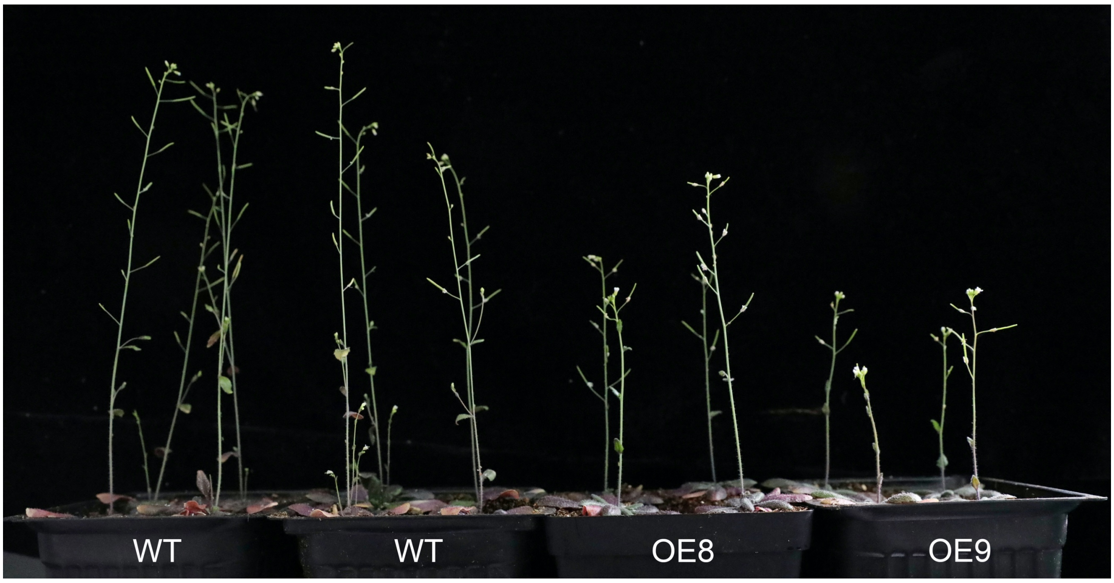

Figure S30: Morphological phenotypes of wild-type and *Arabidopsis* containing the *35S::GhGA2OX8* cDNA construct. WT, OE8 and OE9 represent wild-type and transgenic lines, respectively.

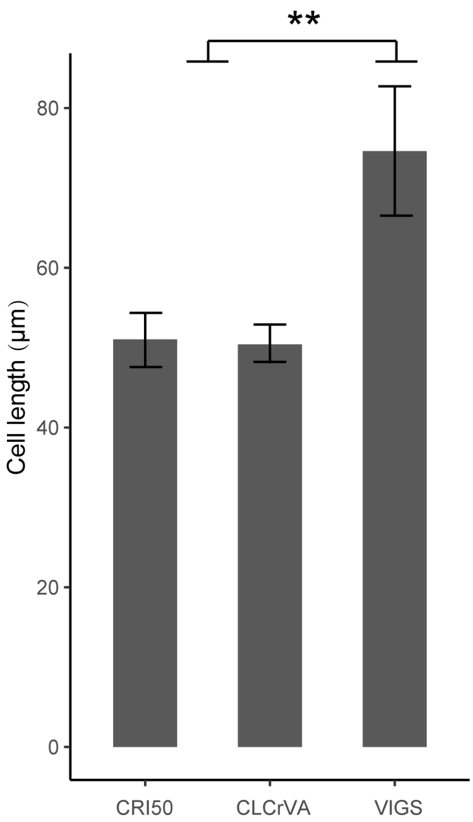

Figure S31: Comparison of cell length of CRI50, CLCrVA and VIGS lines.
**Indicates significance at 0.01.
